# Targeted degradation of aberrant Tau for the discovery of *Pulsatilla chinensis* in Alzheimer’s disease

**DOI:** 10.1186/s13020-025-01276-x

**Published:** 2026-05-20

**Authors:** Lan Deng, Can Yin, Xiaogang Zhou, Chi Feng, Jianming Wu, Xiaobing An, Jianing Mi, Lufen Huang, Dalian Qin, Lu Yu, Ting Chen, Anguo Wu

**Affiliations:** 1 Sichuan Key Medical Laboratory of New Drug Discovery and Drugability Evaluation, Department of Cardiology, School of Pharmacy, The Affiliated Hospital of Southwest Medical University and Key Laboratory of Medical Electrophysiology, Southwest Medical University, Luzhou, 646000 China; 2 School of Pharmaceutical Sciences, China-Pakistan International Science and Technology Innovation Cooperation Base for Ethnic Medicine Development in Huna Province, Hunan University of Medicine, Huaihua, 418000 China; 3 State Key Laboratory of Traditional Chinese Medicine Syndrome, The Second Affiliated Hospital of Guangzhou University of Chinese Medicine, Guangzhou, 510120 Guangdong China; 4 Department of Pharmacy, Jining Medical University, Rizhao, 276500 Shandong China; 5 Institute of Traditional Chinese Medicine Health Industry, China Academy of Chinese Medical Sciences, Nanchang, 330038 China

**Keywords:** Alzheimer’s disease, Tau, *Pulsatilla chinensis* extract, Mitophagy, SH-SY5Y, *Caenorhabditis elegans*

## Abstract

**Background:**

Alzheimer’s disease (AD) is characterized by Tau aggregation, mitochondrial dysfunction, and oxidative stress, yet effective interventions targeting these pathological cascades remain limited. Therapeutic strategies that enhance autophagic and mitophagic clearance, attenuate Tau toxicity, and restore mitochondrial homeostasis are crucial for AD management.

**Methods:**

This study investigated the neuroprotective effects of *Pulsatilla chinensis* extract (PCE) in SH-SY5Y neuronal cells and *Caenorhabditis elegans* (*C. elegans*) models of Tauopathy. Autophagic flux was evaluated by GFP-LC3 puncta formation, LC3-II conversion, and p62 degradation. Mitochondrial function was assessed through reactive oxygen species (ROS) production, mitochondrial membrane potential (MMP), and ultrastructural analysis. The roles of autophagy and mitophagy were examined using 3-methyladenine (3-MA) and the Parkin inhibitor AC220. In *C. elegans*, locomotion, Tau aggregation, oxidative stress, and mitophagosome formation were assessed, and *pink-1* knockdown was used to confirm mitophagy dependence.

**Results:**

PCE significantly enhanced autophagic flux, decreased total and phosphorylated Tau (p-Tau Ser404) levels, and improved neuronal viability. It significantly reduced ROS accumulation, maintained MMP, and preserved mitochondrial morphology under both Tau overexpression and H_2_O_2_-induced oxidative stress. Inhibition of autophagy or Parkin-mediated mitophagy negated these protective effects. In *C. elegans*, PCE ameliorated neuromuscular dysfunction, suppressed Tau inclusions, and reduced oxidative injury, while the loss of *pink-1* abolished its benefits, underscoring the critical role of mitophagy.

**Conclusion:**

PCE exerts potent neuroprotective effects by promoting mitophagy, reducing Tau phosphorylation and aggregation, and restoring mitochondrial integrity. These findings reveal a novel mechanism linking mitochondrial quality control with Tau proteostasis and highlight PCE as a promising natural therapeutic candidate for AD.

**Graphical Abstract:**

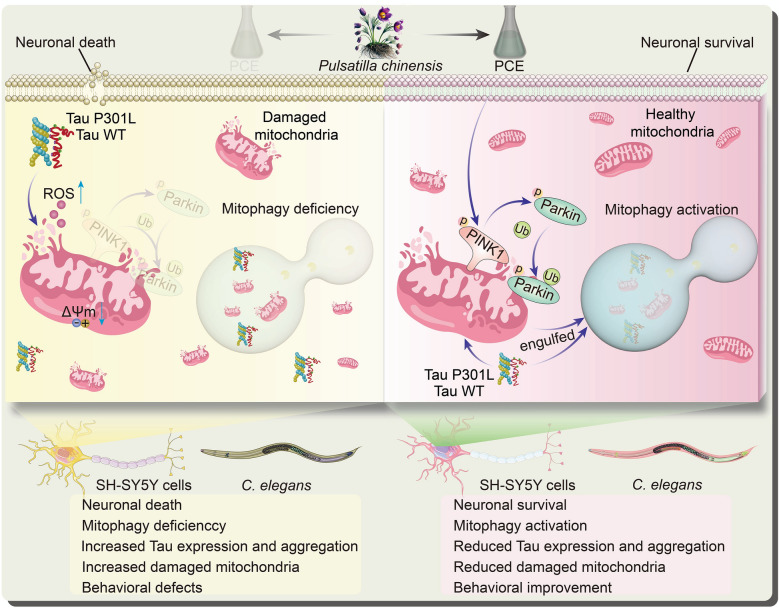

**Supplementary Information:**

The online version contains supplementary material available at 10.1186/s13020-025-01276-x.

## Highlights

- *Pulsatilla chinensis* extract (PCE) activates PINK1/Parkin-mediated mitophagy.
- PCE reduces Tau levels and cytotoxicity and improves motor function in SH-SY5Y cells and* C. elegans*.
- PCE alleviates Tau- or H2O2-induced mitochondrial damage in SH-SY5Y cells and* C. elegans*.
- PCE exerts neuroprotective effects through activating mitophagy in AD.

**Supplementary Information:**

The online version contains supplementary material available at 10.1186/s13020-025-01276-x.

## Introduction

Alzheimer’s disease (AD), the most common form of dementia, is characterized by progressive cognitive decline, synaptic dysfunction, and neuronal loss [1–3]. A defining pathological feature of AD is the abnormal accumulation of hyperphosphorylated Tau protein, which forms neurofibrillary tangles that closely correlate with disease severity [4, 5]. Despite extensive efforts to develop Tau-targeted therapies, no clinically effective interventions have yet succeeded in halting or reversing Tau-driven neurodegeneration [6]. In addition to Tau abnormalities, mitochondrial dysfunction, oxidative stress, and impaired proteostasis interact in a self-reinforcing cycle that accelerates neuronal damage and cognitive decline [7–9].

Growing evidence indicates that autophagy and its selective subtype, mitophagy, are crucial in maintaining neuronal homeostasis by removing damaged organelles and aggregated proteins such as Tau [10, 11]. Impaired autophagic flux and defective mitophagy contribute to the accumulation of dysfunctional mitochondria and pathological Tau species in AD brains [5, 12–14]. Thus, pharmacological activation of autophagy and mitophagy has emerged as a promising strategy for promoting Tau clearance, restoring mitochondrial quality, and alleviating oxidative stress [15–17]. However, discovering safe and effective agents that can concurrently modulate these interconnected pathways remains a major challenge.

Natural products represent a rich reservoir of pharmacologically active molecules with multitarget potential in neurodegenerative diseases [18–23]. *Pulsatilla chinensis* (Bge.) Regel, known as “Bai Tou Weng” in traditional Chinese medicine (TCM), has long been used to treat inflammatory and infectious disorders attributed to “heat” and “toxicity” [24–26]. Modern pharmacological studies have revealed that its saponins and flavonoids exhibit antioxidant, anti-inflammatory, and antiapoptotic activities, suggesting potential neuroprotective relevance [27, 28]. However, the capacity o*f Pulsatilla chinensis* extract (PCE) to modulate autophagic or mitophagic pathways and mitigate Tau-associated pathology has not been explored. In this study, we investigated the neuroprotective and mechanistic effects of PCE in AD models. Using Tau P301L-overexpressing SH-SY5Y cells and transgenic *Caenorhabditis elegans* (*C. elegans*) strains, we evaluated whether PCE could promote mitophagy, reduce pathological Tau accumulation, alleviate oxidative stress, and restore mitochondrial integrity. Our findings demonstrate that PCE enhances PINK1/Parkin-dependent mitophagy, decreases total and phosphorylated Tau levels, and improves neuronal function, revealing its potential as a natural multitarget therapeutic candidate for AD.

## Materials and methods

### Chemicals and reagents

Various chemicals, inhibitors, antibodies, dyes, and plasmids were used in this study. The autophagy inhibitor 3-Methyladenine (3-MA, S276714) was purchased from Selleck Chemicals (Houston, USA), and AC220 (013770477) from Adamas life (Shanghai, China). Bafilomycin A1 (Baf, T6740) and rapamycin (Rap, T1537) were obtained from TargetMol (Boston, USA), while CCCP (555–60-2) was sourced from Yuanye Biotechnology Co., Ltd. (Shanghai, China). LC3 (14,600–1-AP) and p62 (18,420-1-AP) antibodies were purchased from Proteintech (Wuhan, China), Phospho-Tau (Ser404) (20,194) was from Cell Signaling Technology (CST, MA, USA), and GFP (sc-390394) and GAPDH (sc-47724) antibodies were obtained from Santa Cruz Biotechnology (Santa Cruz, CA, USA). Hoechst 33,342 (H8753AABF) was obtained from Jizhi Biochemical Technology Co., Ltd. (Shanghai, China). Propidium iodide (PI) and dihydroethidium (DHE) were purchased from Sigma Chemical Co. (MO, USA). Additionally, tetramethylrhodamine methyl ester (TMRM) for mitochondrial membrane potential (MMP) assessment was sourced from Thermo Fisher Scientific Inc. (CA, USA). Mito-Tracker Red CMXRos and penicillin–streptomycin were from Beyotime Biotechnology Co., Ltd. (Shanghai, China), and 5-fluoro-2′-deoxyuridine (FUDR, F0503) from Aladdin Biochemical Technology Co., Ltd. (Shanghai, China). For plasmid-based experiments, pRK5-EGFP-Tau-P301L (46,908) and pRK5-EGFP-Tau (46,904) plasmids were obtained from Addgene (MA, USA), while Mito-QC plasmid was sourced from Public Protein/Plasmid Library (PPL, Nanjing, China). Lip3000 (TL301-01/02) transfection reagent was purchased from Vazyme Biotech (Nanjing, China).

### Preparation of PCE

PCE was prepared using a traditional ethanol extraction method. Briefly, dried *Pulsatilla chinensis* root was ground into a fine powder. A 75% ethanol solution was used as the solvent for extraction. The powdered material was then mixed with the ethanol at a ratio of 1:10 (w/v) and subjected to reflux extraction at 80 °C for 2 h. The extract was then filtered, and the solvent was evaporated under reduced pressure to obtain the crude extract. The resulting PCE was lyophilized and stored at − 20 °C until use.

### UHPLC-DAD-Q/TOF–MS/MS conditions

The chemical composition of the PCE was analyzed using an Ultra-High Performance Liquid Chromatography (UHPLC) system (Shimadzu, Kyoto, Japan) coupled with a triple quadrupole TOF mass spectrometer (X500R, AB SCIEX, Foster City, CA, USA) equipped with a Duo Spray ionization source. Chromatographic separation was performed on a C18 column (150 mm × 2.1 mm, 1.8 µm particle size, Agilent, USA). The mobile phase consisted of solvent A (0.1% formic acid in water) and solvent B (0.1% formic acid in acetonitrile), with a gradient elution from 5 to 95% B over 20 min, at a flow rate of 0.3 mL/min. Mass spectrometry was carried out in negative ion mode with a capillary voltage of 4.5 kV and a source temperature of 550 °C. The MS parameters were optimized for high sensitivity and resolution. Data acquisition was performed in both full scan mode and product ion mode. The mass range was set from 100 to 1500 m/z for full scan analysis, and collision-induced dissociation (CID) was applied for MS/MS fragmentation. The UV detection was set at 254 nm for chromatographic monitoring. Data analysis was performed using SCIEX OS software (version 3.0).

### Cell culture

Human neuroblastoma SH-SY5Y cells were obtained from the American Type Culture Collection (ATCC, Manassas, VA, USA). SH-SY5Y cells were cultured in Dulbecco’s Modified Eagle Medium (DMEM, Gibco, USA), supplemented with 10% fetal bovine serum (FBS, Procell Life Science & Technology Co., Ltd., Wuhan, China) and 1% penicillin–streptomycin. GFP-LC3 U87 and RFP-GFP-LC3 U87 cells, which express GFP-tagged or RFP/GFP-tagged LC3 for autophagy monitoring, were provided by Dr. Zhu Xiaoming at Macau University of Science and Technology and cultured in alpha Minimum Essential Medium (α-MEM, Gibco, USA) supplemented with 10% FBS and 1% penicillin–streptomycin. All cells were maintained in a humidified incubator at 37 °C with 5% CO_2_.

### MTT assay

Cell viability was assessed using the 3-(4,5-dimethylthiazol-2-yl)-2,5-diphenyltetrazolium bromide (MTT) assay. Cells were seeded in 96-well plates at 1 × 10^4^ cells per well and incubated overnight. After treatment, 10 µL of MTT solution (5 mg/mL) was added to each well and incubated for 4 h. The MTT solution was removed, and the formazan crystals were dissolved in 100 µL DMSO. Absorbance was measured at 570 nm using a microplate reader. Cells transfected with the empty vector (pRK5-EGFP) or treated with the vehicle solvent served as control groups, and their absorbance values were set as 100% viability. Cell viability in all other treatment groups was calculated as a percentage of the control groups.

### Cell transfection

For transient transfection, SH-SY5Y cells were seeded in 6-well plates at a density of 1 × 10^5^ cells per well and incubated overnight to reach 70–80% confluence. The cells were transfected with plasmids using Lip3000 Transfection Reagent (Vazyme Biotech, Nanjing, China) according to the manufacturer’s instructions. Briefly, plasmids or siRNA were diluted in Opti-MEM medium (Invitrogen, Thermo Fisher Scientific Inc., CA, USA), and Lip3000 reagent was mixed with the plasmid solution for 20 min before being added to the cells. After 6 h of incubation, the transfection medium was replaced with a fresh complete medium. Cells were incubated for 24–48 h post-transfection before further experimentation. In this study, pRK5-EGFP-Tau and pRK5-EGFP-Tau P301L were used as the target plasmids to model Tau expression and pathology, and the corresponding pRK5-EGFP was used as the control plasmid to ensure experimental comparability.

### Hoechst/PI staining

Cell death was assessed using Hoechst 33,342 and PI staining. SH-SY5Y cells were seeded in 6-well plates at a density of 1 × 10^5^ cells per well and treated as described. After treatment, cells were washed with phosphate-buffered saline (PBS) and incubated with 10 µg/mL Hoechst 33,342 and 5 µg/mL PI for 15 min at 37 °C in the dark. The cells were then washed with PBS and immediately observed under a fluorescence microscope (Olympus, Japan). Hoechst-stained nuclei were visualized at 405 nm, while PI staining was observed at 595 nm. The blue fluorescence from Hoechst 33,342 indicated the presence of all cells, while the red fluorescence from PI indicated dead cells. This cell death was quantified by calculating the percentage of the number of PI-positive cells to the number of Hoechst-positive cells. Cells transfected with the pRK5-EGFP plasmid or treated with the vehicle solvent served as control groups.

### Western blot analysis

Western blot analysis was performed to assess protein expression levels. After treatment, SH-SY5Y cells or worms were lysed in RIPA buffer (ROO10, Solarbio, China) containing 1% protease inhibitor cocktail (GlpBio, USA) and phosphatase inhibitors (Roche, Germany). The protein concentration was determined using a BCA protein assay kit (Thermo Fisher Scientific Inc., CA, USA). Equal amounts of protein (20–40 µg) were separated by 10–12% Sodium dodecyl-sulfate polyacrylamide gel electrophoresis (SDS-PAGE) and transferred onto polyvinylidene fluoride (PVDF) membranes (PALL, USA). The membranes were blocked with 5% non-fat milk in Tris-buffered saline with 0.1% Tween-20 (TBST) for 1 h at room temperature. Membranes were incubated overnight at 4 °C with primary antibodies, followed by incubation with appropriate HRP-conjugated secondary antibodies (1:2000, MBL, Japan) for 1 h at room temperature. Proteins were detected using the ECL chemiluminescence ChemiDoc Imaging System (Bio-Rad, USA). The band intensities were quantified using ImageJ software (NIH, USA), and the data were normalized to GAPDH expression.

### DHE staining in SH-SY5Y cells

To assess oxidative stress and cell death, Hoechst 33,342 and DHE staining were performed [29]. SH-SY5Y cells were seeded in 6-well plates at a density of 1 × 10^5^ cells per well and treated as described. After treatment, cells were washed with PBS and incubated with 10 µg/mL Hoechst 33,342 for 10 min at 37 °C in the dark to stain the nuclei. Then, cells were incubated with 5 µM DHE for 30 min at 37 °C to detect reactive oxygen species (ROS). Following staining, cells were washed with PBS and immediately observed under a fluorescence microscope (Olympus, Japan). Hoechst-stained nuclei were visualized at 405 nm, and DHE fluorescence indicating ROS production was observed at 610 nm. The relative levels of ROS were quantified by analyzing the fluorescence intensity of DHE staining using ImageJ software (NIH, USA). The ratio of DHE-positive cells to Hoechst-positive cells was used to determine the extent of oxidative stress-induced damage. Cells transfected with the pRK5-EGFP plasmid or treated with the vehicle solvent served as control groups.

### TMRM staining

MPP/Δψm was assessed using Hoechst 33,342 and TMRM staining [30]. SH-SY5Y cells were seeded in 6-well plates at a density of 1 × 10^5^ cells per well and treated as described. After treatment, cells were washed with PBS and incubated with 10 µg/mL Hoechst 33,342 for 10 min at 37 °C to stain the nuclei. Following nuclear staining, cells were incubated with 100 nM TMRM for 20 min at 37 °C. TMRM accumulates in healthy mitochondria with high membrane potential, emitting red fluorescence. After staining, cells were washed with PBS and immediately observed under a fluorescence microscope (Olympus, Japan). Hoechst-stained nuclei were visualized at 405 nm, while TMRM fluorescence indicating MMP was observed at 590 nm. Loss of MMP was indicated by a decrease in TMRM fluorescence intensity, and the ratio of TMRM-positive cells to Hoechst-positive cells was quantified using ImageJ software (NIH, USA). Cells transfected with the pRK5-EGFP plasmid or treated with the vehicle solvent served as control groups.

### Mito-tracker staining

Mitochondrial integrity was assessed using Hoechst 33,342 and Mito-Tracker staining [31]. SH-SY5Y cells were seeded in confocal plates at a density of 1 × 10^5^ cells per well and treated as described. Cells were allowed to adhere to the wall overnight, and after the experimental treatments, cells were washed using PBS and stained with 10 µg/mL Hoechst 33,342 for 10 min at 37 °C to label nuclei. Following nuclear staining, cells were incubated with 100 nM Mito-Tracker Red CMXRos for 30 min at 37 °C. Mito-Tracker specifically stains mitochondria, allowing for visualization of mitochondrial morphology. After staining, cells were washed with PBS and observed using a Zeiss confocal microscope (Zeiss, Germany). Hoechst-stained nuclei were visualized at 405 nm, and Mito-Tracker Red fluorescence was observed at 590 nm. Mitochondrial length was analyzed using ImageJ software (NIH, USA), measuring the longest mitochondrial axis in each image to assess mitochondrial morphology. This analysis involved binarization of images, skeletonization, and quantification of mitochondrial morphology parameters, including branch length and the number of individuals. Cells transfected with the pRK5-EGFP plasmid or treated with the vehicle solvent served as control groups.

### GFP-RFP-LC3 fluorescence analysis

Autophagic flux was evaluated using GFP-RFP-LC3 fluorescence analysis in RFP-GFP-LC3 U87 cells [32]. Cells were seeded in 6-well plates at a density of 1 × 10^5^ cells per well and treated as described. After treatment, cells were washed with PBS and fixed with 4% paraformaldehyde for 15 min at room temperature. The fixed cells were then washed with PBS and mounted with an antifade mounting medium containing DAPI (Beyotime Biotechnology, China) to stain the nuclei. The cells were observed using a Zeiss confocal microscope (Zeiss, Germany), and GFP and RFP fluorescence were detected at 488 nm and 590 nm, respectively. GFP fluorescence reflects the formation of autophagosomes, while RFP fluorescence indicates both autophagosomes and autolysosomes. The number of GFP and RFP puncta was quantified using ImageJ software (NIH, USA). The ratio of GFP (autophagosomes) to RFP (autolysosomes) puncta was calculated to evaluate autophagic flux. Cells treated with the vehicle solvent served as the control group.

### Mito-QC mitophagy assay

Mitophagy was assessed using the Mito-QC assay in SH-SY5Y cells. Cells were seeded in 6-well plates at a density of 1 × 10^5^ cells per well and transfected with Mito-QC plasmid using Lip3000 reagent, according to the manufacturer's instructions. After 24 h of transfection, cells were treated with the indicated compounds for the specified time. Following treatment, cells were fixed with 4% paraformaldehyde for 15 min and mounted with antifade mounting medium containing DAPI. Mitophagy was analyzed using a fluorescence microscope (Olympus, Japan), with GFP fluorescence detected at 488 nm and mCherry fluorescence observed at 590 nm. The GFP/mCherry fluorescence ratio was quantified using ImageJ software (NIH, USA) to evaluate the mitophagic flux. Cells treated with the vehicle solvent served as the control group.

### Co-localization of mitochondria with autophagosomes

The co-localization of mitochondria with autophagosomes was evaluated using GFP-LC3 U87 cells. Cells were seeded in confocal plates at a density of 1 × 10^5^ cells per well and treated as described. After treatment, cells were washed with PBS and stained with Mito-Tracker Red CMXRos for 30 min at 37 °C to label mitochondria. Following mitochondrial staining, cells were fixed with 4% paraformaldehyde for 15 min at room temperature. The cells were then washed with PBS and mounted with antifade mounting medium containing DAPI to stain the nuclei. Co-localization of mitochondria and autophagosomes was visualized using a Zeiss confocal microscope (Zeiss, Germany). GFP-LC3 puncta (autophagosomes) were detected at 488 nm, and Mito-Tracker Red (mitochondria) fluorescence was observed at 590 nm. The extent of co-localization was analyzed using ImageJ software (NIH, USA), with the number of co-localized puncta quantified to assess mitophagy activity. Cells treated with the vehicle solvent served as the control group.

### *C. elegans* strains and culture

The following *C. elegans* strains were used in this study: BR5271[rab-3p::F3(delta)K280 I277P I380P + myo-2p::mCherry], BR5270[rab-3p::F3(delta)K280 + myo-2p::mCherry], KAE112[myo-3p::human tau (0N4R;V337M)::unc-54 3'UTR + vha-6p::mCherry::unc-54 3'UTR]., SJ4103[myo-3::GFP(mit)], BC12921[rCes T12G3.1::GFP + pCeh361], DA2123[lgg-1p::GFP::lgg-1p::GFP::lgg-1 + rol-6 (su1006)], MH215[lgg-1p::mCherry::GFP::lgg-1 + rol-6], and SJZ42[rgef-1p::tomm-20::Rosella]. All strains were obtained from the *C. elegans* Genetics Center (CGC). *C. elegans* were maintained at 20 °C on Nematode Growth Medium (NGM) plates seeded with *Escherichia coli* (*E. coli*) OP50 as a food source. The worms were cultured under standard conditions, with a 12-h light/dark cycle. To synchronize the populations, worms were treated with a bleaching procedure, and L1 larvae were collected and placed onto fresh NGM plates for subsequent experiments.

### Swimming body bend assays

Swimming body bend assays were performed using BR5270 and the normal control BR5271 *C. elegans* strains. Synchronized L1 larvae of both strains were placed onto NGM plates seeded with *E. coli* OP50 and allowed to grow to the young adult stage at 20 °C. For the swimming assay, individual worms were placed in a 10 cm Petri dish filled with M9 buffer (22 mM KH_2_PO_4_, 42 mM Na_2_HPO_4_, 85 mM NaCl, 1 mM MgSO_4_) and gently tapped to initiate swimming, allowing the nematodes to acclimatize to the environment for 1 min. The swimming behavior was observed for 20 s, and the number of body bends (a complete curvature of the body from head to tail) was counted. A body bend was defined as a visible arc in the worm’s body. Each condition was tested with at least 30 worms, and the assay was performed in triplicate. The swimming speed and body bend frequency were compared between the treated and control groups (vehicle solvent) to evaluate the effects of treatments on motor function.

### Food-sensing ability

Food-sensing ability, indicating the behavioral performance of worms, was assessed using BR5270 and the normal control BR5271 *C. elegans* strains [33, 34]. Synchronized L1 larvae of both strains were cultured on NGM plates seeded with *E. coli* OP50 and allowed to grow to the young adult stage at 20 °C. To assess food-sensing behavior, individual worms were placed onto NGM plates without bacteria (starvation plates) for 1 h to induce a feeding response. Then, the worms were transferred to fresh NGM plates with *E. coli* OP50 placed in a quarter section of the plate. The worms’ movement toward the food source was monitored and recorded for 10 min. The number of worms in contact with the bacterial lawn was counted, and the response rate was calculated as the percentage of worms that moved towards the food. Slowing rate = (locomotion rate in the absence of food − locomotion rate in the presence of food) / locomotion rate in the absence of food. The assay was performed in triplicate for each strain, with at least 30 worms per trial. Differences in food-sensing ability were compared between the experimental and control groups (vehicle solvent) to evaluate the effect of treatments on chemosensory behavior.

### Egg-laying assays

Egg-laying assays were conducted using the KAE112 *C. elegans* strain [35]. Synchronized L1 larvae were cultured on NGM plates seeded with *E. coli* OP50 and allowed to grow to the young adult stage at 20 °C. For the egg-laying assay, individual young adult worms were transferred onto fresh NGM plates and allowed to lay eggs for 4 h at 20 °C. The number of eggs laid by each worm was counted under a stereomicroscope (Leica, Germany) at the end of the incubation period. After 24 h, each worm was transferred to a new NGM medium, and the number of eggs laid by each worm in each dish was counted until the reproductive ability of the worm was lost. The assay was performed in triplicate for each condition, with at least 20 worms per trial. The average number of eggs laid per worm was calculated and compared between experimental and control groups (vehicle solvent) to evaluate the impact of treatments on reproductive behavior and egg-laying capacity.

### Paralysis assays

Paralysis assays were conducted using the KAE112 *C. elegans* strain to assess the neuroprotective effects of treatments [36]. Synchronized L1 larvae were cultured on NGM plates seeded with *E. coli* OP50 and allowed to grow to the young adult stage at 20 °C. For the paralysis assay, young adult worms were exposed to either control conditions or experimental treatments for 48 h. After exposure, worms were transferred to NGM plates containing 50 mM FUDR to inhibit egg laying. The worms were then placed at 20 °C and observed for paralysis, which was defined as the inability to move in response to gentle prodding. Paralysis was scored as follows: (1) paralyzed, when the worm was immobile; (2) moving, when the worm could respond to gentle tapping; and (3) unresponsive, when the worm did not move at all. The percentage of paralyzed worms in each group was recorded at the specified time points. Each condition was tested with at least 30 worms, and the assay was performed in triplicate. The results were compared between experimental and control groups (vehicle solvent) to determine the effect of the experimental treatment on the neurodegenerative phenotype.

### Lifespan analysis

Lifespan analysis was conducted using the KAE112 *C. elegans* strain to evaluate the effects of treatments on longevity [36]. Synchronized L1 larvae were cultured on NGM plates seeded with *E. coli* OP50 and allowed to develop to the young adult stage at 20 °C. For lifespan analysis, adult worms were transferred onto fresh NGM plates and treated with either the control or experimental compounds. The worms were monitored daily for survival, and dead worms were counted. The criterion for death was defined as a lack of response to gentle prodding. The lifespan of each group was determined by recording the number of days until 50% of the worms in the group were dead. At least 100 worms were used per group, and the experiment was repeated three times. The data were analyzed using Kaplan–Meier survival curves, and statistical differences between experimental and control groups (vehicle solvent) were determined using the log-rank test.

### DHE staining in *C. elegans*

DHE staining was performed to assess ROS levels in BR5271 and its normal control BR5270 *C. elegans* strains [20]. Synchronized L1 larvae were cultured on NGM plates seeded with *E. coli* OP50 and allowed to develop to the young adult stage at 20 °C. For ROS detection, the young adult worms were incubated with 10 µM DHE dissolved in M9 buffer for 60 min at 20 °C. After incubation, the worms were washed three times with M9 buffer to remove excess dye. The stained worms were then mounted on a glass slide and observed under a fluorescence microscope (Zeiss, Germany) with excitation at 485 nm and emission at 590 nm. The fluorescence intensity was quantified using ImageJ software, and the average fluorescence per worm was calculated. The results were compared between the experimental and control groups (vehicle solvent) to evaluate the effect of treatments on ROS levels.

### Measurement of mitochondrial integrity in *C. elegans*

Mitochondrial integrity in the SJ4103 *C. elegans* strain, which expresses GFP-labeled mitochondria in body wall muscle cells, was assessed without the need for additional mitochondrial staining [29, 37]. Synchronized L1 larvae were cultured on NGM plates seeded with *E. coli* OP50 and allowed to develop to the young adult stage at 20 °C. For analysis, young adult worms were mounted on a glass slide, and mitochondrial integrity was observed directly using a fluorescence microscope (Zeiss, Germany). The GFP signal was excited at 488 nm, and the emission was captured at 509 nm to visualize the mitochondria. The worms treated with the vehicle solvent were severed as the control group.

### Autophagy detection in *C. elegans*

Autophagy detection was performed in *C. elegans* strains expressing specific autophagy markers, including SQST-1:GFP (BC12921), LGG-1:GFP (DA2123), and tandem-tagged mCherry::GFP::LGG-1 (MAH215). The BC12921 strain expresses SQST-1::GFP, the homolog of the mammalian autophagy receptor p62, while DA2123 expresses LGG-1::GFP, a marker for autophagosome formation, homologous to mammalian LC3 [38]. The MAH215 strain carries the tandem-tagged mCherry::GFP::LGG-1 fusion protein, allowing the detection of autophagosome maturation [39]. Worms were cultured at 20 °C until they reached the L4 stage, then synchronized by egg laying and maintained at the L1 stage. L1 worms were treated with test compounds for 48 h on NGM plates. To eliminate any influence from egg-laying, the treatment period lasted for 48 h. Following treatment, the worms were washed three times in M9 buffer to remove excess bacteria and transferred to slides for imaging. Fluorescence microscopy was performed using a Leica DM6B system. For the BC12921 strain, the GFP signal from SQST-1 was quantified using ImageJ software. In the DA2123 strain, autophagosome formation was assessed by counting GFP::LGG-1 puncta per unit area. In the MAH215 strain, mCherry::GFP fluorescence was analyzed to quantify autophagosome formation and maturation, providing a comprehensive assessment of autophagy in the worms. The worms treated with the vehicle solvent were severed as the control group.

### Mitophagy detection in *C. elegans*

Mitophagy was assessed in *C. elegans* using the SJZ42 strain, which expresses neuron-specific, mitochondria-localized Rosella (foxEx3 [rgef-1p::tomm-20::Rosella]), a pH-sensitive fluorescent biosensor [40, 41]. L4-stage worms were synchronized by egg-laying and treated with PCE or CCCP for 48 h. After treatment, worms were washed with M9 buffer and mounted on microscope slides for imaging. Fluorescence microscopy was performed using a Leica DM6B system. Mitophagy was evaluated by analyzing the GFP and DsRed fluorescence signals, where a decrease in the GFP/DsRed fluorescence ratio indicates mitophagy induction. Images were processed to quantify changes in mitochondrial dynamics. The worms treated with the vehicle solvent were severed as the control group.

### RNAi in *C. elegans*

RNA interference (RNAi) was performed in *C. elegans* using the BR5270 and KAE112 strains. For RNAi knockdown, L4-stage worms were fed HT115 bacteria carrying the *pink-1* RNAi construct. The worms were synchronized by egg-laying and transferred to NGM plates containing the RNAi bacteria. After 48 h of incubation at 20 °C, worms were washed with M9 buffer to remove residual bacteria, and then processed for further assays. The RNAi treatment was confirmed by assessing phenotypic changes, including behavioral assays, ROS levels, and p-Tau level to evaluate the impact of *pink-1* knockdown. The HT115 bacteria-fed worms treated with the vehicle solvent were severed as the control group.

### Statistical analysis

Statistical analysis was performed using GraphPad Prism (version 9.0). Data are presented as mean ± standard error of the mean (SEM). Comparisons between two groups were performed using unpaired Student's t-test, Comparisons between two groups were made using unpaired Student's t-test. A one-way Analysis of Variance (ANOVA) followed by Tukey's post-hoc test was used to assess differences among various groups. Kaplan–Meier survival curves were analyzed using the log-rank test. A p-value of < 0.05 was considered statistically significant. All experiments were conducted at least three times.

## Results

### PCE induces autophagy and reduces Tau P301L cytotoxicity

**Fig. 1:**
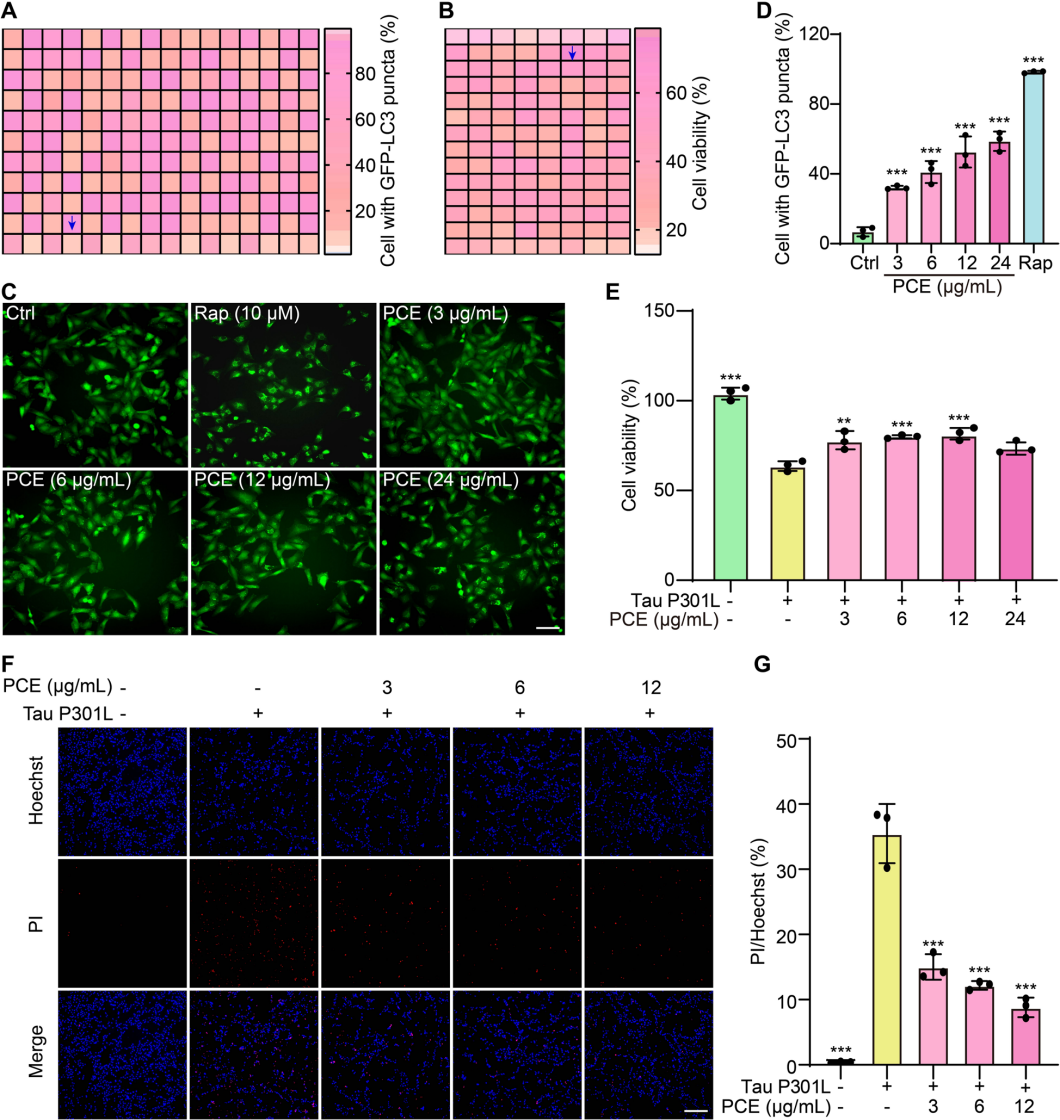
PCE is identified as a potential autophagy inducer that reduces Tau P301L-induced cytotoxicity. **A**, **B** Representative heatmaps showing the percentage of cells with GFP-LC3 puncta in GFP-LC3 U87 cells (**A**) and cell viability in pRK5-EGFP-Tau-P301L (Tau P301L)-transfected SH-SY5Y cells (**B**) after treatment with various nature medicine extracts. **C** Representative fluorescence images of GFP-LC3 U87 cells treated with Ctrl, Rap (10 μM), or increasing concentrations of PCE (3, 6, 12, 24 μg/mL), showing the formation of GFP-LC3 puncta. Magnification: 10 × ; scale bar: 200 μm. **D** Quantification of cells with GFP-LC3 puncta under the indicated conditions. **E** Cell viability of Tau P301L-expressing SH-SY5Y cells treated with different concentrations of PCE. **F** Representative fluorescence images of Tau P301L-expressing SH-SY5Y cells stained with Hoechst (blue) and PI (red) in the presence or absence of PCE. Magnification: 10 × ; scale bar: 200 μm. **G** Quantification of cell death based on the PI/Hoechst ratio. Data are presented as mean ± SEM. Significant difference is denoted as *p < 0.05, **p < 0.01, and ***p < 0.001

To explore potential autophagy-enhancing agents that could mitigate Tau-related neurotoxicity, we first screened a panel of natural medicine extracts in GFP-LC3 U87 cells and Tau P301L-overexpressing SH-SY5Y cells. Among the tested candidates, PCE emerged as a prominent autophagy inducer and displayed a protective effect against Tau P301L cytotoxicity. Heatmap analyses revealed that PCE treatment significantly increased the proportion of cells with GFP-LC3 puncta in GFP-LC3 U87 cells (Fig. 1A and Fig. S1A) and enhanced overall cell viability in Tau P301L-expressing SH-SY5Y cells (Fig. 1B and Fig. S1B). Consistent with these findings, fluorescence imaging demonstrated a clear dose-dependent increase in GFP-LC3 puncta formation when cells were treated with 3 to 24 μg/mL PCE, resembling the autophagy-inducing effect of the positive control Rap (Fig. 1C, D). Parallel experiments using Tau P301L-overexpressing SH-SY5Y cells further confirmed that PCE treatment significantly improved cell viability and morphology, counteracting the cytotoxic impact of Tau P301L aggregation (Fig. 1E and Fig. S2). This protective effect was corroborated by Hoechst/PI dual staining, which showed a marked reduction in the proportion of PI-positive, nonviable cells following PCE administration (Fig. 1F, G). Further UHPLC-DAD-Q/TOF–MS/MS analysis showed that the main components in PCE were caftaric acid, cichoric acid, beesioside Q, pulsatilloside E, and so on (Fig. S3, 4 and Table S1). Collectively, these results highlight the capacity of PCE to activate autophagy and attenuate Tau P301L-induced cell death, establishing a foundation for its potential therapeutic application in AD.

### PCE reduces pathogenic Tau expression, phosphorylation, and aggregation in SH-SY5Y cells

**Fig. 2:**
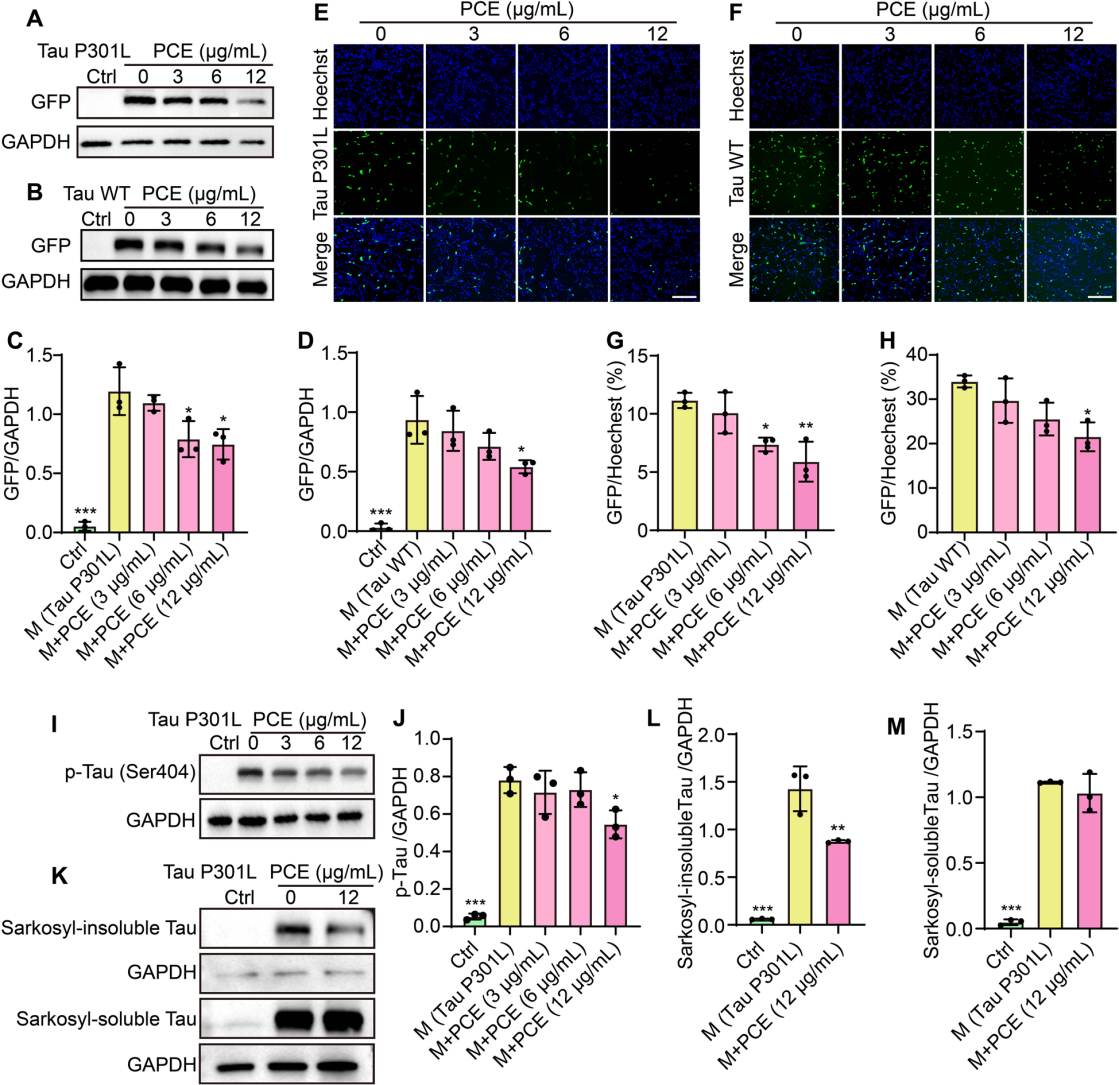
PCE reduces Tau expression, pathogenic phosphorylation, and aggregation in SH-SY5Y cells. **A**, **B** Western blots showing GFP-tagged Tau P301L and Tau WT expression levels after treatment with increasing concentrations of PCE (3, 6, and 12 µg/mL). Full-length Western blotting images were provided in Fig. S6. **C**, **D** Quantification of GFP expression normalized to GAPDH. **E**, **F** Representative fluorescence images showing GFP-tagged Tau P301L and Tau WT expression in SH-SY5Y cells under different PCE concentrations, with nuclei stained by Hoechst. Magnification: 10 × ; scale bar: 200 µm. **G**, **H** Quantification of the GFP fluorescence intensity relative to Hoechst, indicating Tau expression changes upon PCE treatment. **I**, **J** Western blot and quantification of p-Tau (Ser404) levels in Tau P301L-transfected cells treated with different concentrations of PCE. **K** Western blot analysis showing Sarkosyl-insoluble and -soluble Tau fractions in Tau P301L cells with or without PCE (12 μg/mL) treatment. **L**, **M** Quantification of insoluble and soluble Tau normalized to GAPDH. Data are presented as mean ± SEM. Significant difference is denoted as *p < 0.05, **p < 0.01, ***p < 0.001

To further evaluate the impact of PCE on pathologically relevant Tau species, we assessed how PCE treatment influences both mutant (Tau P301L) and wild-type (Tau WT) Tau expression levels in SH-SY5Y cells. Western blot analyses revealed that PCE at concentrations of 3, 6, and 12 µg/mL elicited a dose-dependent reduction in GFP-tagged Tau P301L and Tau WT protein levels relative to untreated controls (Fig. 2A-D). Consistent with the biochemical data, fluorescence microscopy of cells co-stained with Hoechst validated the decrease in intracellular Tau signal following PCE treatment. Representative images indicate that cells expressing GFP-tagged Tau P301L (Fig. 2E, G) or Tau WT (Fig. 2F, H) exhibited noticeably lower GFP fluorescence after incubation with PCE. To gain further mechanistic insight, we next examined whether PCE affects pathogenic Tau phosphorylation and aggregation. Western blotting demonstrated that PCE significantly reduced the levels of p-Tau (Ser404), a pathogenic modification strongly associated with neurofibrillary tangle formation (Fig. 2I–J). Furthermore, analysis of Sarkosyl-fractionated lysates revealed that PCE treatment markedly decreased the accumulation of insoluble Tau aggregates (Fig. 2K–M), suggesting enhanced clearance of aggregated Tau. Consistent with these findings, fluorescence images showed that PCE markedly diminished the number and intensity of GFP-positive Tau P301L inclusions in SH-SY5Y cells, indicating suppression of Tau aggregation and a more diffuse cytoplasmic distribution of Tau (Fig. S5). Together, these results demonstrate that PCE not only decreases total and phosphorylated Tau levels but also inhibits the formation of insoluble aggregates, highlighting its potential to mitigate multiple pathological features of Tau toxicity.

### *PCE reduces oxidative damage in Tau P301L-expressing or H*_*2*_*O*_*2*_*-induced SH-SY5Y cells*

**Fig. 3:**
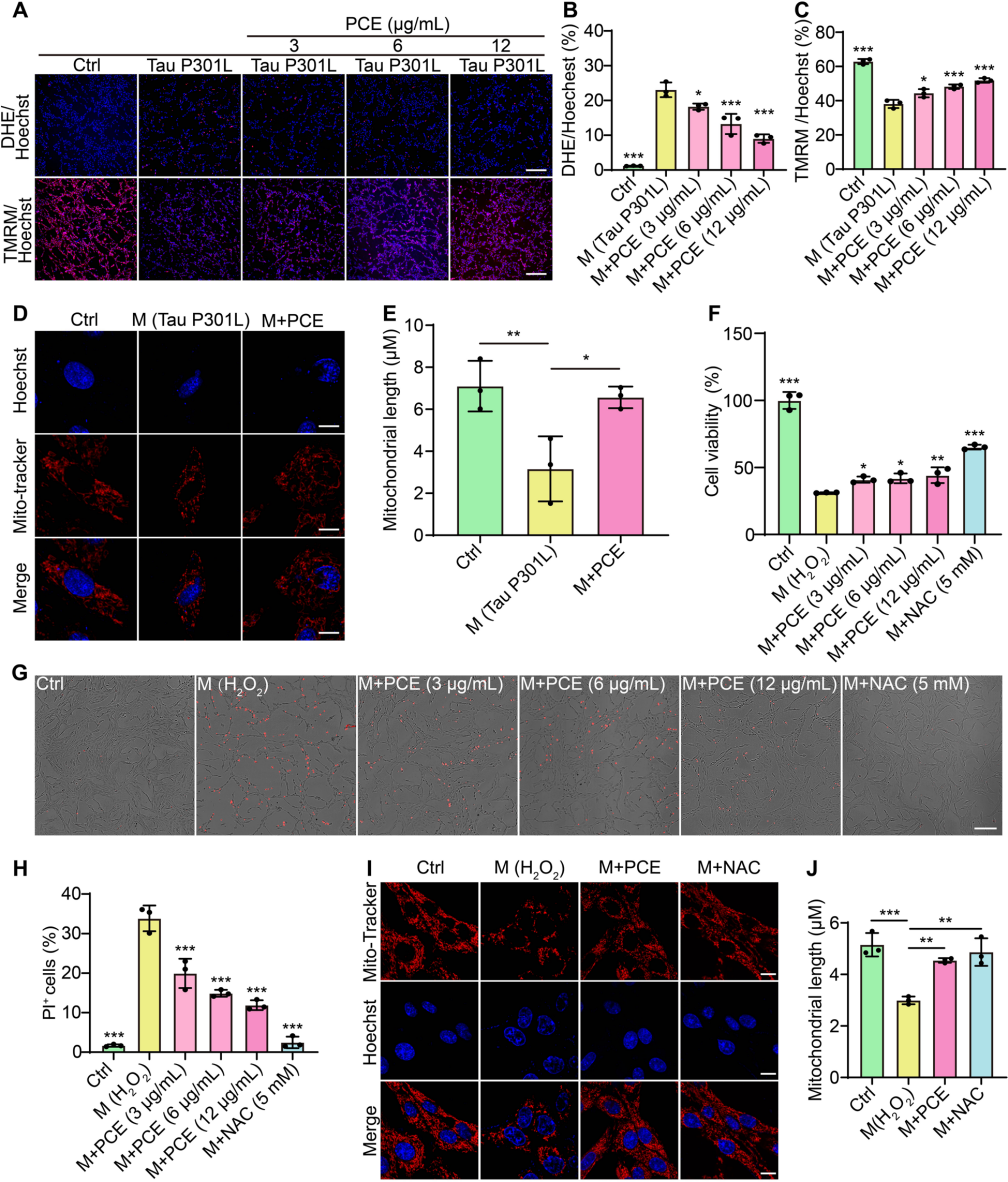
PCE alleviates oxidative stress and preserves mitochondrial morphology in Tau P301L-expressing and H_2_O_2_-treated SH-SY5Y cells. **A** Representative fluorescence images of SH-SY5Y expressing Tau P301L and treated with PCE (3, 6, or 12 μg/mL). Cells are stained with DHE (upper panels) to detect ROS and TMRM (lower panels) to assess MMP. Magnification: 10 × ; scale bar: 200 µm. **B**, **C** Quantification of DHE-positive cells (B) and TMRM-positive (C) cells under the indicated conditions. **D** Representative confocal images of Hoechst (blue) and Mito-Tracker (red)-stained SH-SY5Y cells under Ctrl, Tau P301L, and Tau P301L plus PCE conditions. Magnification: 63 × ; scale bar: 10 µm. **E** Quantification of mitochondrial length in SH-SY5Y cells. **F** Cell viability determined by MTT assay in SH-SY5Y cells exposed to H_2_O_2_ (200 μM) alone or co-treated with PCE (12 μg/mL) or NAC (5 mM). **G** Bright-field images of PI-stained SH-SY5Y cells showing cell death under Ctrl, H_2_O_2_, and co-treatment conditions. Magnification: 10 × ; scale bar: 200 µm. **H** Quantification of PI-positive SH-SY5Y cells. **I** Representative confocal images of Mito-Tracker-stained SH-SY5Y cells under Ctrl, H_2_O_2_, and co-treatment conditions. Magnification: 63 × ; scale bar:10 µm. **J** Quantification of mitochondrial length under the indicated treatments. Data are presented as mean ± SEM. Significant difference is denoted as *p < 0.05, **p < 0.01, ***p < 0.001

To determine whether PCE’s protective effects against aberrant Tau extend to the oxidative stress often associated with neurodegenerative processes, we next examined intracellular ROS levels, MMP, and overall mitochondrial health in Tau P301L-overexpressing SH-SY5Y cells. As shown in Fig. 3A, B, DHE staining revealed that Tau P301L expression markedly increased the number of DHE-positive cells compared to controls, indicating elevated ROS production, while PCE treatment significantly reduced the elevated ROS levels induced by Tau P301L overexpression. To assess mitochondrial integrity, we employed TMRM staining to monitor MMP. The presence of Tau P301L resulted in a loss of TMRM fluorescence, reflecting impaired MMP. Notably, PCE treatment at increasing concentrations counteracted this effect, leading to a significant recovery of TMRM staining intensity (Fig. 3A, C). Confocal imaging with Mito-Tracker further substantiated these improvements. Whereas Tau P301L expression caused an apparent fragmentation and shortening of mitochondria, PCE treatment preserved or restored mitochondrial morphology and length to more elongated and intact forms (Fig. 3D, E). In addition to reducing endogenous ROS, PCE conferred resistance against exogenous oxidative stressors. When cells were exposed to H_2_O_2_ (200 μM), cell viability dramatically declined. However, co-treatment with PCE or the known antioxidant NAC rescued cell viability to a substantial degree (Fig. 3F). PI staining confirmed these protective effects, showing that PCE reduced the proportion of non-viable cells in an H_2_O_2_-stressed environment, paralleling the antioxidant action of NAC (Fig. 3G, H). Confocal imaging with Mito-Tracker staining revealed that PCE, like NAC, preserved mitochondrial morphology under oxidative stress conditions, preventing the severe mitochondrial fragmentation and shortening induced by H_2_O_2_ (Fig. 3I, J). Collectively, these findings indicate that PCE not only reduces ROS levels and maintains MMP but also restores mitochondrial morphology and bolsters cell viability under oxidative challenge in Tau P301L-expressing or H_2_O_2_-induced SH-SY5Y cells.

### PCE protects against combined oxidative and Tau-induced stress

**Fig. 4:**
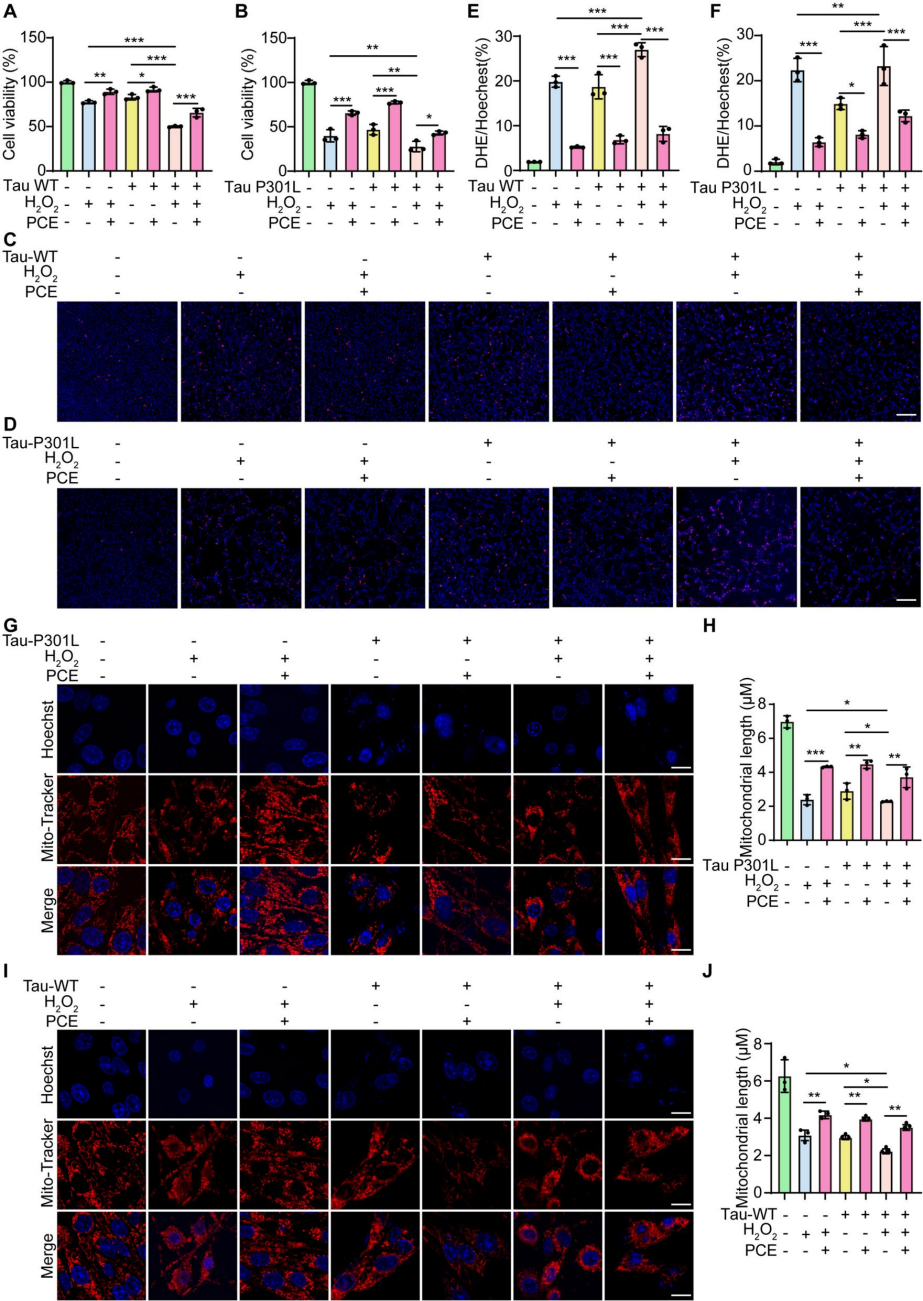
PCE protects SH-SY5Y cells against combined H_2_O_2_- and Tau-induced oxidative and mitochondrial damage. **A**, **B** Cell viability measured by MTT assay in SH-SY5Y cells overexpressing P301L Tau or Tau WT, treated with H_2_O_2_ (200 μM) and/or PCE (12 μg/mL). **C**, **D** Representative DHE fluorescence images showing intracellular ROS levels under combined H_2_O_2_ and Tau-induced stress. Blue: Hoechst (nuclei); Red: DHE (ROS). Magnification: 10 × ; scale bar: 200 µm. **E**, **F** Quantification of ROS levels normalized to Hoechst (%). **G**, **I** Representative confocal images of mitochondrial morphology in Tau P301L and Tau WT-overexpressing cells exposed to H_2_O_2_ with or without PCE treatment. Mitochondria were stained with Mito-Tracker, nuclei with Hoechst. Magnification: 63 × ; scale bar: 10 µm. **H**, **J** Quantification of mitochondrial length. Data are presented as mean ± SEM. Significant difference is denoted as *p < 0.05, **p < 0.01, ***p < 0.001

To further investigate whether PCE could protect against combined oxidative and Tau-induced cellular insults, we examined its effects in SH-SY5Y cells simultaneously challenged with H_2_O_2_ and Tau overexpression. MTT assays revealed that while either H_2_O_2_ exposure or Tau overexpression alone reduced cell viability, the combination produced a more pronounced cytotoxic effect, particularly in Tau P301L-transfected cells (Fig. 4A, B). PCE treatment significantly rescued cell viability under these dual stress conditions. ROS production was markedly elevated in both WT and P301L Tau cells upon H_2_O_2_ treatment, as indicated by strong DHE fluorescence. The combined presence of H_2_O_2_ and Tau further exacerbated ROS accumulation, highlighting a synergistic effect. Notably, PCE substantially reduced ROS levels in both groups, restoring them toward control levels (Fig. 4C-F). Since Tau aggregation and oxidative stress are closely linked to mitochondrial dysfunction, we next assessed mitochondrial morphology. In both WT and P301L Tau cells exposed to H_2_O_2_, mitochondria exhibited severe fragmentation and loss of network integrity. Treatment with PCE preserved elongated mitochondrial structures, significantly increasing average mitochondrial length compared to untreated cells (Fig. 4G–J). Together, these results demonstrate that PCE effectively counteracts the synergistic damage induced by both Tau overexpression and oxidative stress, protecting mitochondrial integrity and improving neuronal cell survival.

### PCE activates mitophagy in SH-SY5Y cells

**Fig. 5:**
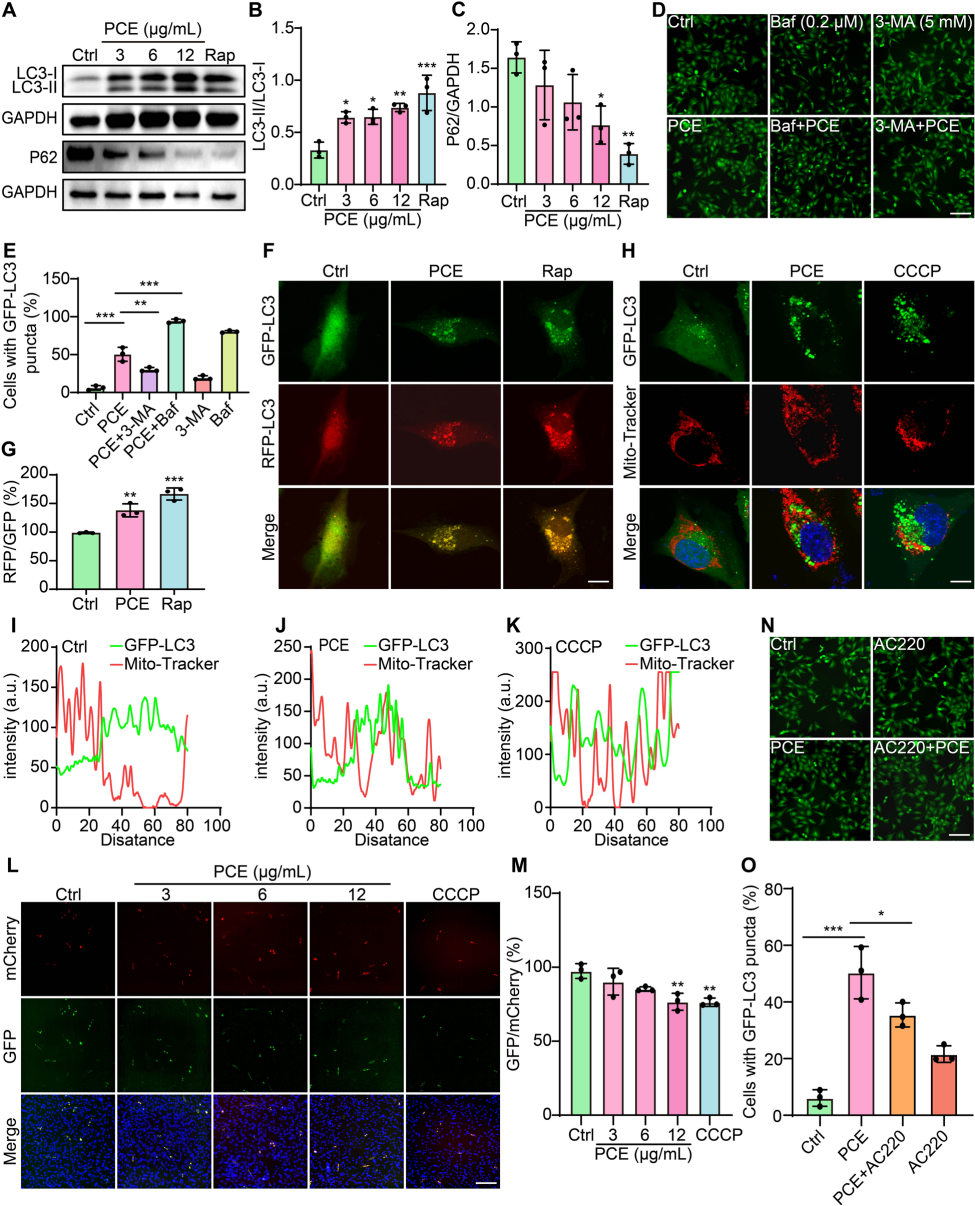
PCE activates mitophagy in vitro. **A** Representative Western blot showing LC3 and p62 levels in SH-SY5Y cells treated with Ctrl, PCE (3, 6, 12 µg/mL), or Rap. GAPDH serves as a loading control. Full-length Western blots are shown in Fig. S7. **B**, **C** Quantification of LC3-II/LC3-I ratio and p62/GAPDH ratio. **D** Representative fluorescence images of GFP-LC3 U87 cells under Ctrl, PCE, Baf, or 3-MA treatments, alone or in combination. Magnification: 10 × ; scale bar: 200 µm. **E** Quantification of GFP-LC3 puncta in GFP-LC3 U87 cells under the indicated treatments. **F** Representative confocal images of RFP-GFP-LC3 U87 cells showing autophagosome formation following Ctrl, PCE, or Rap treatments. Magnification: 63 × ; scale bar: 10 µm. **G** Quantification of RFP/GFP fluorescence ratio in RFP-GFP-LC3 U87 cells. **H** Representative confocal images of GFP-LC3 U87 cells co-stained with Mito-Tracker after Ctrl, PCE, or CCCP (10 μM) treatment. Yellow signals in merged images indicate mitochondrial co-localization with LC3 puncta. Magnification: 63 × ; scale bar: 10 µm. **I–K** Line-scan intensity profiles of GFP-LC3 and Mito-Tracker fluorescence from representative cells under Ctrl (I), PCE (J), and CCCP (K) treatments. **L** Representative fluorescence images of SH-SY5Y cells expressing the dual-fluorescent mitophagy reporter Mito-QC following Ctrl, PCE (3, 6, 12 µg/mL), or CCCP treatment. Hoechst stains nuclei (blue). Magnification: 10 × ; scale bar: 200 µm. **M** Quantification of GFP/mCherry fluorescence ratio in Mito-QC-expressing SH-SY5Y cells. **N** Representative fluorescence images of GFP-LC3 U87 cells treated with Ctrl, PCE, AC220 (a Parkin inhibitor, 1 μM), or the combination of PCE and AC220. Magnification: 10 × ; scale bar: 200 µm. **O** Quantification of cells containing GFP-LC3 puncta under the indicated treatments. Data are presented as mean ± SEM. Significant differences are denoted as *p < 0.05, **p < 0.01, ***p < 0.001

To further elucidate the mechanism underlying PCE’s protective actions, we investigated whether PCE could promote the selective degradation of mitochondria through mitophagy. Western blot analyses revealed that PCE treatment at concentrations of 3, 6, and 12 µg/mL significantly increased the LC3-II/LC3-I ratio while reducing p62 levels, mirroring the effects of Rap and indicating enhanced autophagic flux (Fig. 5A-C). Pharmacological inhibition experiments further confirmed this phenomenon that the autophagy inhibitors 3-MA prevented the formation of GFP-LC3 puncta and Baf further promoted the accumulation of GFP-LC3 puncta triggered by PCE (Fig. 5D, E). To directly visualize autophagosomal formation and flux, we employed RFP-GFP-LC3-expressing U87 cells. PCE and Rap treatments robustly increased the RFP-LC3/GFP-LC3 puncta ratio (Fig. 5F, G). This elevated ratio indicates the successful autophagosome maturation into autolysosomes, underscoring PCE’s capacity to enhance autophagic degradation. Next, we assessed whether PCE activate mitophagy. Confocal imaging of GFP-LC3 U87 cells co-stained with Mito-Tracker revealed that PCE and mitophagy inducer CCCP promoted the colocalization of GFP-LC3 puncta with mitochondria (Fig. 5H). Line-scan analyses of fluorescence intensity profiles confirmed these observations, showing spatial overlap between LC3 and Mito-Tracker signals in PCE-treated cells (Fig. 5I-K). In SH-SY5Y cells expressing a dual fluorescent mitophagy reporter (Mito-QC), PCE reduced the GFP/mCherry ratio, indicative of enhanced mitophagic flux and mitochondrial degradation, comparable to CCCP treatment (Fig. 5L, M). To further delineate the mechanism of PCE-induced mitophagy, we tested the role of Parkin, a key regulator of mitochondrial quality control. Application of AC220, a Parkin inhibitor, attenuated PCE-induced GFP-LC3 puncta formation, suggesting that PCE’s mitophagic effect is at least partly Parkin-dependent (Fig. 5N, O). Taken together, these results demonstrate that PCE enhances mitophagy in SH-SY5Y cells.

### *PCE inhibits Tau-induced cytotoxicity *via* mitophagy induction*

**Fig. 6:**
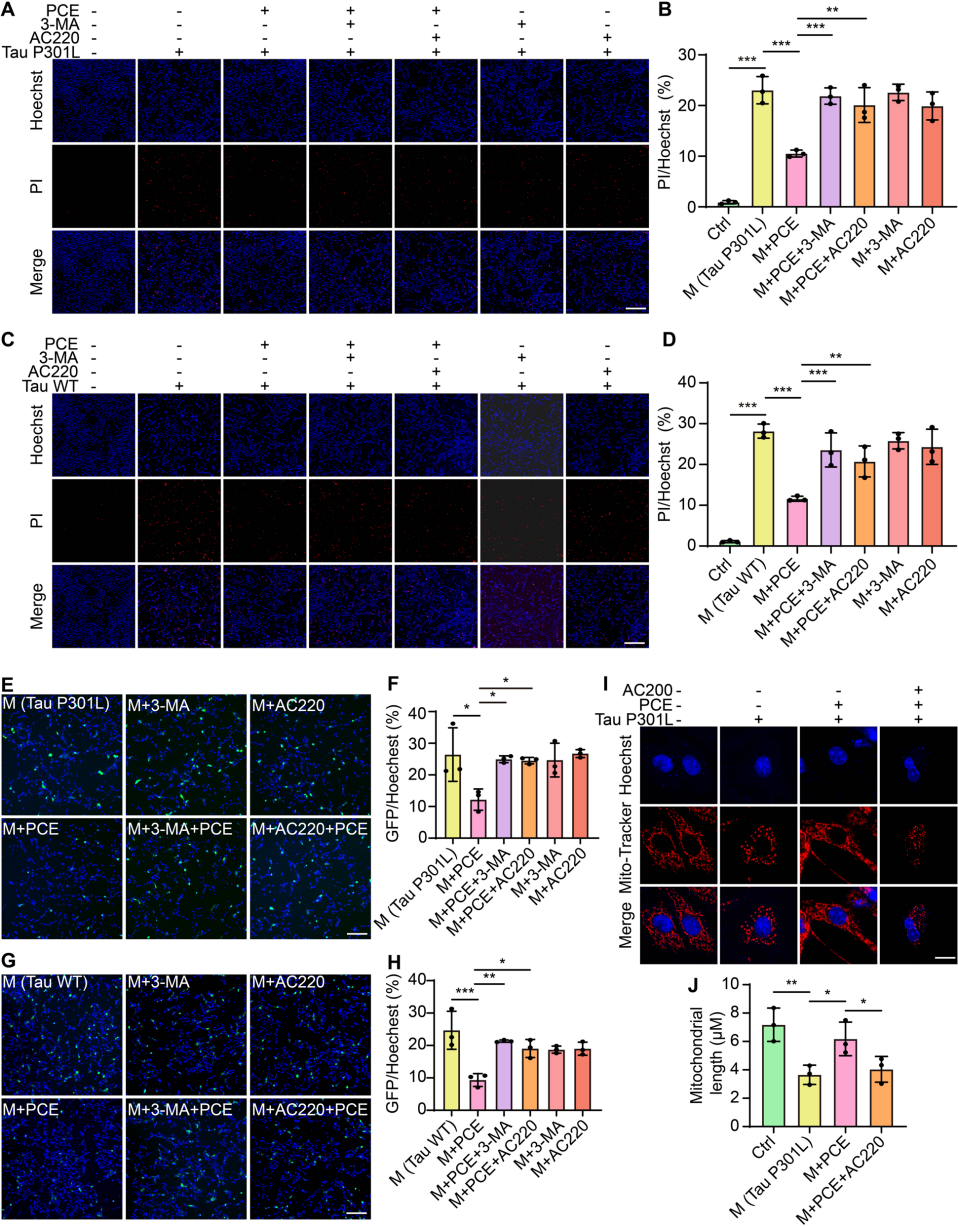
Inhibition of mitophagy reverses PCE-mediated protective effects against Tau-induced cytotoxicity. **A**, **C** Representative fluorescence images of SH-SY5Y cells expressing Tau P301L (A) or Tau WT (C) treated with PCE (12 µg/mL), 3-MA (2 mM), or AC220 (1 µM) alone or in combination, and stained with Hoechst (blue) and PI(red). Magnification: 10 × ; scale bar: 200 µm. **B**, **D** Quantification of PI-positive cells (PI/Hoechst ratio). **E**, **G** Representative fluorescence images of SH-SY5Y cells expressing GFP-tagged Tau P301L (E) or Tau WT (G) treated with PCE, 3-MA, or AC220 alone or in combination, and stained with Hoechst. Magnification: 10 × ; scale bar: 200 µm. **F**, **H** Quantification of GFP/Hoechst ratio in SH-SY5Y cells. **I** Representative confocal images of SH-SY5Y cells expressing Tau P301L treated with PCE, AC220, or their combination, stained with Mito-Tracker (red) and Hoechst (blue). Magnification: 63 × ; scale bar: 10 µm. **J** Quantification of mitochondrial length under the indicated treatments. Data are presented as mean ± SEM. Significance is denoted as *p < 0.05, **p < 0.01, ***p < 0.001.

To understand whether PCE-mediated mitophagy plays a pivotal role in protecting against Tau-induced cytotoxicity, we investigated the consequences of mitophagy inhibition on PCE’s protective effects. As shown in Fig. 6A-D, PCE treatment significantly reduced the number of PI-positive cells in both Tau P301L- and Tau WT-expressing SH-SY5Y cells. However, this protective effect was markedly diminished when cells were co-treated with 3-MA or AC220, as evidenced by the increased proportion of PI-positive cells. These findings suggest that PCE’s cytoprotective action is at least partially dependent on autophagic and Parkin-mediated pathways. Furthermore, fluorescence imaging revealed that PCE effectively reduced GFP-tagged Tau P301L and Tau WT signals (Fig. 6E-H), consistent with our previous observations. Notably, when autophagy or Parkin-mediated mitophagy was inhibited, the ability of PCE to clear Tau was substantially weakened, demonstrating that the pro-mitophagic activity of PCE is crucial for its capacity to alleviate Tau pathology. To directly visualize the role of mitophagy, we employed Mito-Tracker staining in Tau P301L-expressing SH-SY5Y cells. Confocal imaging showed that PCE improved mitochondrial integrity, as indicated by elongated and organized mitochondrial networks, while inhibition of Parkin with AC220 largely reversed this effect, resulting in shorter and more fragmented mitochondria (Fig. 6I, J). Taken together, these results demonstrate that PCE’s protective effects against Tau-induced cytotoxicity are mediated by its ability to induce mitophagy. By facilitating the selective removal of damaged mitochondria and reducing intracellular Tau accumulation, PCE exerts a dual action that ultimately enhances neuronal cell survival.

### PCE exhibits neuroprotective effects in transgenic *C. elegans* tauopathy models

**Fig. 7:**
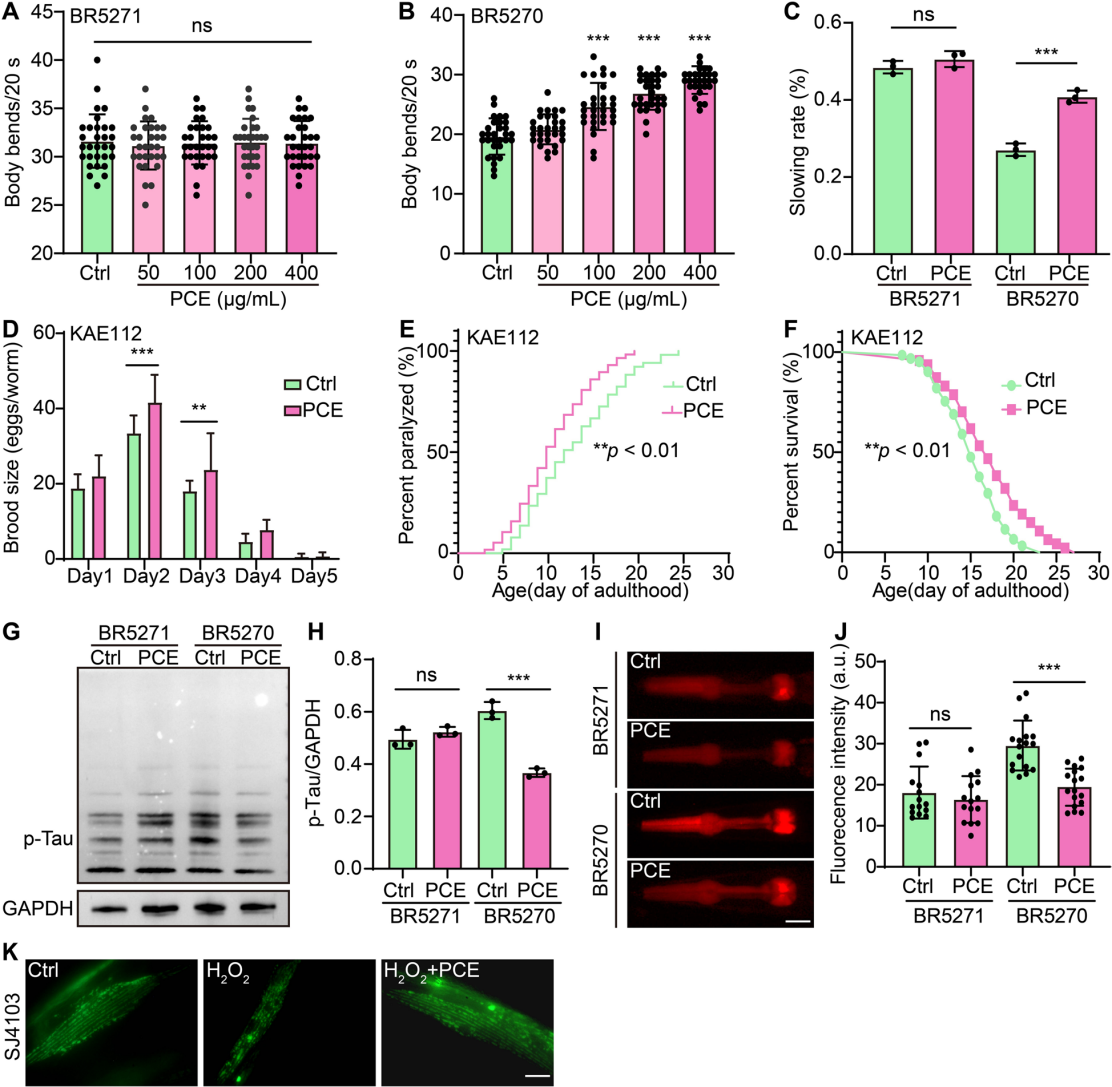
PCE reduces pathogenic tau aggregation and improves physiological function in transgenic *C. elegans* tauopathy models. **A**, **B** Quantification of body bends in BR5271 and BR5270 worms expressing human tau fragments treated with PCE (50, 100, 200, 400 μg/mL). BR5270 (*byIs161*) expresses a pro-aggregation tau fragment, whereas BR5271 (*byIs162*) expresses a non-aggregating variant as a control. **C** Quantification of the slowing rate in BR5271 and BR5270 worms under PCE treatment. **D** Brood size of KAE112 worms after five days of PCE treatment during adulthood. **E** Quantification of locomotor impairment in KAE112 worms over time with or without PCE. **F** Survival curves of KAE112 worms treated with or without PCE. **G** Representative immunoblot images showing p-Tau levels in BR5271 and BR5270 worms following PCE treatment. **H** Quantification of p-Tau band intensity from immunoblots in BR5271 and BR5270 strains. Full-length blots are shown in Fig. S8. **I** Representative DHE-stained fluorescence images of BR5271 and BR5270 worms illustrating ROS levels under PCE treatment. Magnification: 20 × ; scale bar: 100 μm. **J** Quantification of DHE fluorescence intensity corresponding to ROS levels. **K** Representative confocal images showing the mitochondrial morphology in H_2_O_2_-induced SJ4103 worms treated with or without PCE. Magnification: 100 × ; scale bar: 10 μm. Data are presented as mean ± SEM. Significant difference is denoted as ns (not significant), *p < 0.05, **p < 0.01, ***p < 0.001

To extend our findings beyond mammalian cell culture and investigate the in vivo relevance of PCE, we tested its neuroprotective capacity in multiple transgenic *C. elegans* tauopathy models. In BR5271 and BR5270 worms, which express human tau fragments, PCE administration at concentrations ranging from 50 to 400 μg/mL enhanced neuromuscular function, as evidenced by increased body bend. Notably, BR5270 exhibits a pro-aggregation tau fragment and thus displays more pronounced motor deficits, while PCE treatment significantly ameliorated these impairments compared to the non-aggregating control strain BR5271 (Fig. 7A, B). Correspondingly, the slowing rate observed under tau pathology conditions was substantially reduced following PCE exposure (Fig. 7C). Additional tests in KAE112 worms, a model that progressively exhibits locomotor deficits and reduced brood size, further underscored the protective effects of PCE. Daily treatment for five days of adulthood maintained brood size, mitigated locomotor impairment, and improved survival outcomes (Fig. 7D-F). Immunoblot analyses revealed that PCE treatment also reduceed phosphorylated tau (p-Tau) levels in BR5270 worms but not in BR5271 worms, indicating a direct effect on pathogenic tau aggregation (Fig. 7G, H). To investigate whether these protective outcomes were associated with oxidative stress modulation, we examined intracellular ROS levels. DHE staining indicated a notable decrease in ROS fluorescence intensity in BR5270 worms but not in BR5271 worms treated with PCE, reflecting reduced oxidative damage (Fig. 7I, J). Finally, mitochondrial morphology assessments in H_2_O_2_-induced SJ4103 worms demonstrated that PCE treatment restored mitochondrial integrity (Fig. 7K). Collectively, these results highlight PCE’s ability to mitigate tau-induced pathogenic events in *C. elegans*, improving neuromuscular performance, reproductive fitness, locomotion, lifespan, and mitochondrial health.

### PCE activates mitophagy in *C. elegans*

**Fig. 8:**
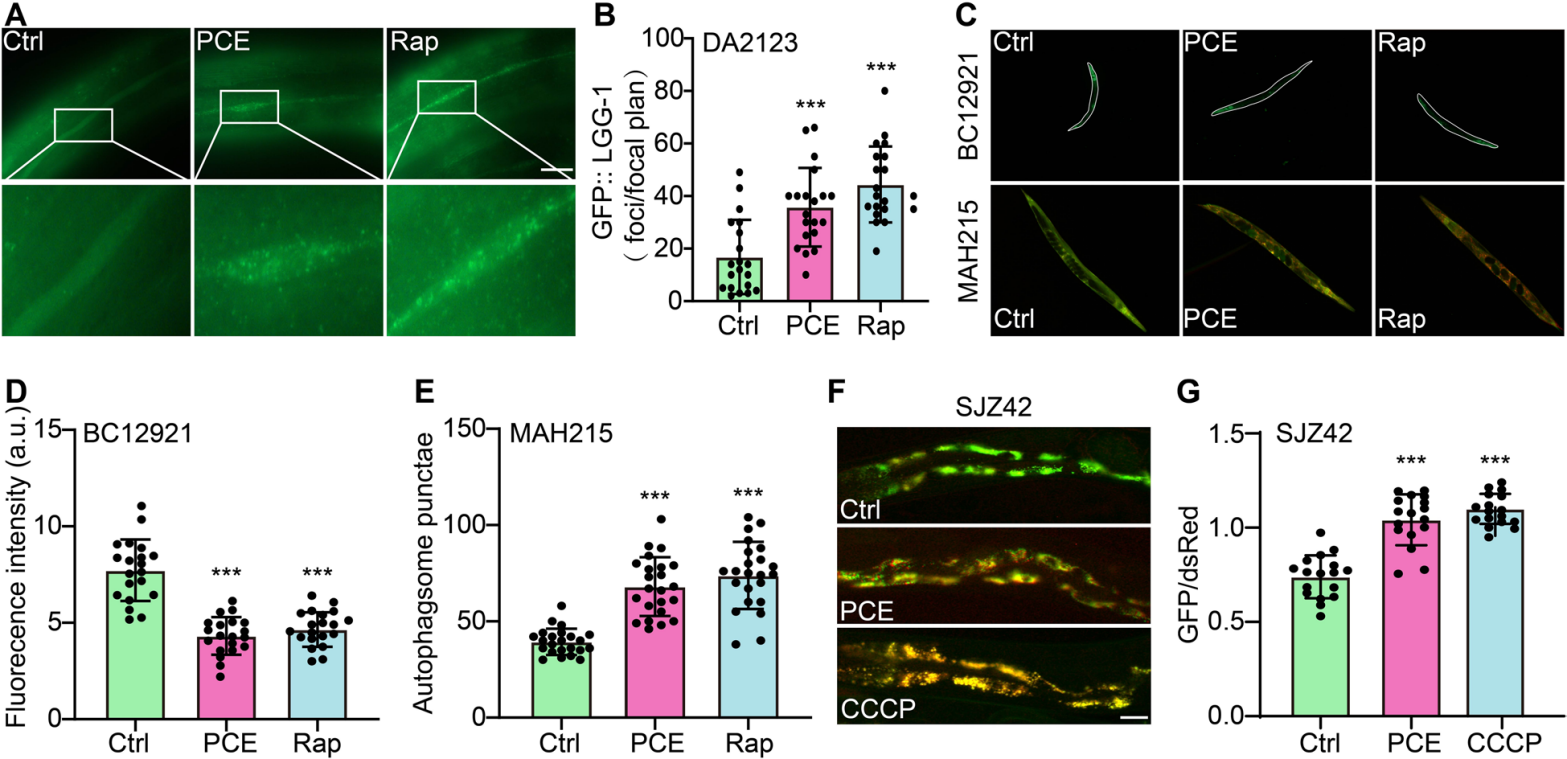
PCE activates mitophagy in *C. elegans*. **A** Representative fluorescence images of DA2123 worms (expressing GFP::LGG-1 as an autophagosome marker) under Ctrl, PCE, and Rap (20 µM) treatment. Boxed regions are shown at higher magnification below. Magnification: 100 × ; scale bar: 10 μm. **B** Quantification of GFP::LGG-1 puncta per focal plane in DA2123 worms under the indicated treatments. **C** Representative fluorescence images of BC12921 (*SQST*-*1*::*GFP*) and MAH215 (*lgg-1p*::mCherry::GFP::lgg-1) worms treated with Ctrl, PCE, or Rap. Magnification: 10 × ; scale bar: 200 μm. **D** Quantification of *SQST*-*1*::*GFP* fluorescence intensity (arbitrary units, AU) in BC12921 worms under different treatments. **E** Quantification of autophagosome puncta in MAH215 worms under Ctrl, PCE, or Rap treatment. **F** Representative fluorescence images of SJZ42 worms carrying a dual-fluorescence reporter (GFP/dsRed) for mitophagy monitoring, treated with Ctrl, PCE, or CCCP (10 μM). Magnification: 20 × ; scale bar: 100 μm. **G** Quantification of GFP/dsRed fluorescence ratio in SJZ42 worms under different treatments. Data are presented as mean ± SEM. Significant difference is denoted as ns, *p < 0.05, **p < 0.01, ***p < 0.001

To determine whether the mitophagy-inducing effects of PCE observed in mammalian cells also occur in vivo, we examined various *C. elegans* transgenic models that report autophagic and mitophagic events. In DA2123 worms expressing GFP:: LGG-1, an established autophagosome marker, PCE treatment substantially increased the number of GFP::LGG-1 puncta per focal plane, similar to Rap (Fig. 8A, B). This result suggests that PCE enhances autophagosomal formation within the nematode intestinal cells. To further confirm autophagic flux, we analyzed BC12921 worms, which express SQST-1::GFP, and MAH215 worms, harboring the dual fluorescent mCherry::GFP::LGG-1 reporter. PCE treatment reduced SQST-1::GFP fluorescence intensity, indicative of increased cargo degradation, while also elevating autophagosome counts in MAH215 worms to levels comparable to Rap-treated controls (Fig. 8C-E). These results strongly support that PCE promotes not only the formation but also the turnover of autophagosomes in *C. elegans*. Finally, to assess whether PCE-induced autophagy encompasses the selective removal of damaged mitochondria, we employed the SJZ42 strain, which carries a dual fluorescence reporter system (GFP/dsRed) for mitophagy. Notably, PCE treatment lowered the GFP/dsRed ratio to an extent similar to CCCP, reflecting enhanced mitochondrial clearance (Fig. 8F, G). Collectively, these findings demonstrate that PCE is capable of activating mitophagy in vivo.

### *PCE exhibits neuroprotective effects *via* mitophagy induction in transgenic C. elegans tauopathy models*

**Fig. 9:**
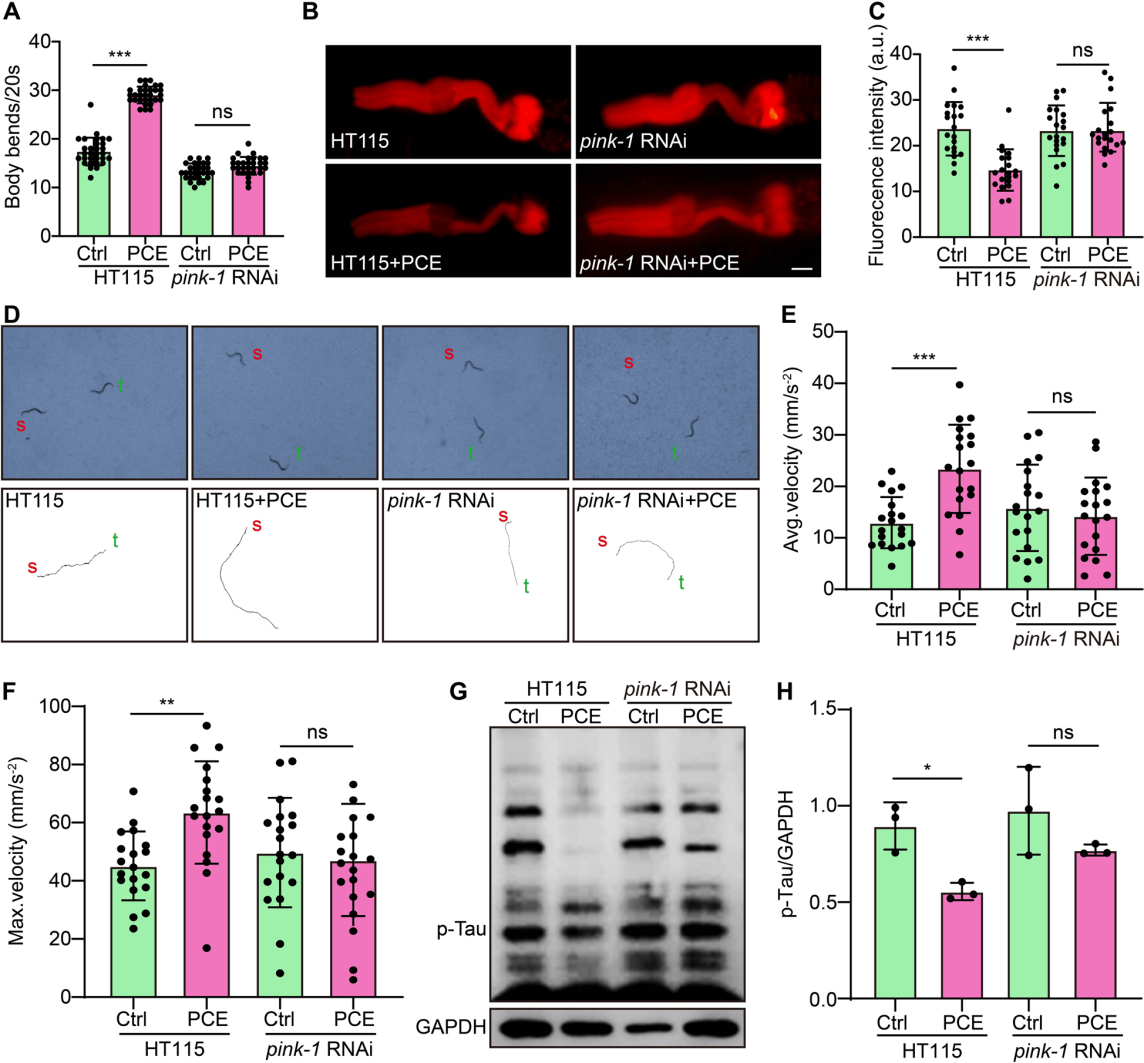
PCE-mediated improvements in tau-associated phenotypes require *pink-1* in *C. elegans*. **A** Quantification of locomotor activity, represented as body bends per 20 s, in BR5270 worms fed with Ctrl bacteria (HT115) or *pink-1* RNAi bacteria, with or without PCE treatment. **B** Representative fluorescence images of DHE-stained BR5270 worms under the indicated conditions. Magnification: 20 × ; scale bar: 100 µm. **C** Quantification of DHE fluorescence intensity (arbitrary units, AU) reflecting ROS levels. **D** Representative locomotion tracks of individual worms from each experimental group (red “s” = start position; green “t” = tracked trajectory). **E**, **F** Quantification of average and maximum velocity (mm/s^2^) in KEA112 worms subjected to HT115 or *pink-1* RNAi feeding, with or without PCE treatment. **G** Representative immunoblot showing p-Tau expression in BR5270 worms fed with HT115 or *pink-1* RNAi bacteria, with or without PCE treatment. GAPDH serves as a loading control. Full-length blots are provided in Fig. S9. **H** Densitometric analysis of p-Tau levels normalized to GAPDH. Data are presented as mean ± SEM. Significant difference is denoted as ns, *p < 0.05, **p < 0.01, ***p < 0.001

To further explore the mechanism underlying PCE’s neuroprotective actions, we examined whether the mitophagy-related kinase *pink-1* is required for PCE-mediated improvements in tauopathy phenotypes. As shown in Fig. 9A, PCE treatment significantly enhanced locomotor function, reflected by increased body bends, in BR5270 worms under normal conditions (HT115 bacteria feeding). However, this improvement was abolished when *pink-1* was knocked down by RNAi feeding, indicating that *pink-1* is essential for PCE’s protective effect on motor behavior. In line with these findings, PCE reduced ROS levels, as detected by DHE staining, but this antioxidant effect was also lost in *pink-1* RNAi-treated worms (Fig. 9B, C). Analysis of worm locomotion trajectories further confirmed that PCE treatment improved movement parameters in KAE112 worms, evident as increased average and maximum velocities under control conditions (Fig. 9D-F). Again, these enhancements disappeared upon *pink-1* depletion. Consistent with our earlier observations, PCE treatment reduced p-Tau levels in BR5270 worms, and this effect was significantly diminished in worms with *pink-1* knockdown (Fig. 9G, H). Together, these data demonstrate that PCE’s ability to mitigate tau-induced defects, reduce oxidative stress, and lower p-Tau accumulation in *C. elegans* depends on the *pink-1*-mediated mitophagy pathway.

## Discussion

**Fig. 10:**
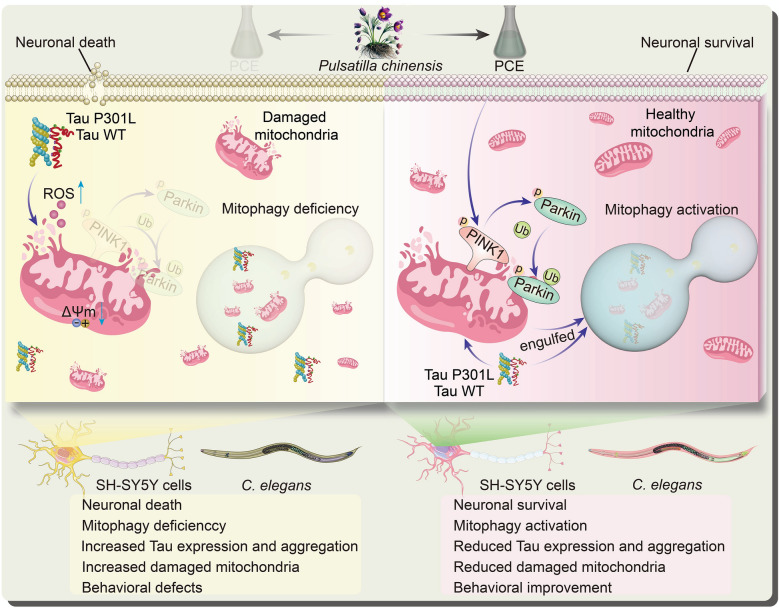
Schematic illustration of the proposed mechanism by which PCE protects against Tau-induced neurotoxicity. Overexpression of Tau WT or Tau P301L causes mitochondrial depolarization, increased ROS production, defective PINK1/Parkin-mediated mitophagy, accumulation of damaged mitochondria, neuronal death, and behavioral defects in SH-SY5Y cells and C. elegans. PCE activates PINK1/Parkin-dependent mitophagy, promotes the engulfment and clearance of damaged mitochondria, reduces Tau expression and aggregation, preserves mitochondrial homeostasis, and ultimately improves neuronal survival and behavioral outcomes

This study identifies PCE as a promising natural agent capable of simultaneously inducing autophagy and mitigating Tau-induced cytotoxicity, thus contributing to a more integrated therapeutic strategy against AD. By employing a screening approach that prioritized both autophagic enhancement and reductions in Tau-related toxicity, we discovered that PCE exerts its protective effects through multiple mechanisms. Our findings indicate that PCE not only facilitates selective mitochondrial clearance via mitophagy, but also reduces total and phosphorylated Tau levels, suppresses Tau aggregation, preserves mitochondrial integrity, and mitigates oxidative stress, ultimately improving cell viability and physiological parameters in both mammalian neuronal cells and transgenic *C. elegans* models of Tauopathy (Fig. 10).

A key finding of the present study is the capacity of PCE to enhance mitophagy and alleviate several interrelated pathogenic processes characteristic of neurodegenerative disorders. Although Tau pathology is a central feature of AD, the disease is also defined by mitochondrial dysfunction, chronic oxidative stress, impaired proteostasis, and progressive neuronal degeneration [42, 43]. Previous studies have shown that interventions targeting autophagy can facilitate the clearance of misfolded proteins, including Tau [44, 45]. However, the current data add a valuable insight that effective treatments may require not only the removal of aggregated proteins but also the restoration of organellar homeostasis. By increasing LC3-II levels, reducing p62, and promoting GFP-LC3 puncta formation, PCE demonstrates classical signs of autophagy induction. Importantly, we further demonstrated that PCE significantly decreased pathogenic p-Tau (Ser404) and reduced Sarkosyl-insoluble Tau aggregates, indicating that PCE promotes both Tau degradation and the suppression of its aggregation. These findings provide new mechanistic evidence that PCE modulates multiple Tau species relevant to AD pathology.

More notably, the co-localization of LC3-positive structures with mitochondria and the requirement of PINK1/Parkin for PCE’s protective effects suggest that enhanced mitophagy is essential for its ability to reduce Tau toxicity. This aligns with emerging views that mitochondrial quality control is integral to maintaining neuronal health and may be critical in combatting AD-related pathology [46, 47]. Many known autophagy inducers, such as Rap, have shown promise in preclinical models of protein aggregation diseases, including AD [48, 49]. However, most studies have focused on the removal of pathogenic proteins without directly addressing the underlying mitochondrial defects that perpetuate oxidative stress and neuronal vulnerability. PCE’s unique advantage may lie in its capacity to act at the nexus of proteostasis and mitochondrial maintenance. By concurrently decreasing Tau phosphorylation and aggregation while improving mitochondrial quality control, PCE achieves dual regulation of protein and organellar homeostasis. By reducing ROS levels, stabilizing MMP, and preventing the fragmentation of mitochondrial networks, PCE ensures that neurons have a healthier intracellular environment in which abnormal Tau species are efficiently cleared. This dual functionality could confer more durable neuroprotection than approaches that only partially address the multifactorial nature of AD.

These findings also align with recent reports showing that Tau aggregates can accumulate within mitochondria, disrupting respiratory function, reducing ATP synthesis, and impairing mitophagy [12, 50, 51]. Pathogenic Tau species such as P301L have been shown to associate with mitochondrial membranes and inhibit Parkin translocation, thereby blocking mitophagic clearance. The observed PCE-induced activation of PINK1/Parkin-mediated mitophagy in our study may therefore promote the selective removal of Tau-containing mitochondria, alleviating mitochondrial stress and restoring bioenergetic homeostasis. Moreover, the PCE-mediated reduction in p-Tau (Ser404) and insoluble aggregates suggests that clearance of Tau-laden mitochondria may be mechanistically linked to the observed decrease in intracellular Tau burden. This mechanism could represent a critical link between the degradation of aberrant Tau and the preservation of mitochondrial function, reinforcing the concept that targeting mitochondrial quality control can indirectly facilitate Tau clearance and neuronal survival.

The use of transgenic *C. elegans* tauopathy models allowed us to validate the in vitro observations in a whole-organism context. Although nematodes cannot replicate the complexity of mammalian neural networks or the full clinical spectrum of AD, they present a convenient and evolutionarily conserved system for studying fundamental aspects of Tau biology and neurodegeneration [52, 53]. In these models, PCE improved neuromuscular function, maintained brood size, and prolonged survival, a strong indication that the PCE’s effects extend beyond simple cytoprotection in cell culture. The reduction in Tau inclusions and p-Tau levels observed in PCE-treated worms further supports its role in mitigating pathological Tau aggregation in vivo. Reductions in p-Tau levels and diminished ROS fluorescence intensity in the worm models mirror the outcomes seen in mammalian cells, enhancing the credibility of our findings. Importantly, the loss of PCE’s protective effect under *pink-1* RNAi conditions in worms highlights the conserved importance of mitophagy across species, strengthening the notion that this pathway is central to PCE’s effects.

Despite these promising results, several questions and limitations must be addressed to advance the translational potential of PCE. First, PCE is a crude natural extract containing a complex mixture of bioactive compounds. While this complexity might enable synergistic interactions that enhance its overall efficacy, it also complicates the identification of the key constituents responsible for the observed neuroprotective effects. Future efforts should focus on bioassay-guided fractionation and chemical characterization to pinpoint the principal active molecules and elucidate their mechanisms. Such endeavors could include advanced spectroscopic analyses, metabolomic profiling, and structure–activity relationship studies. Once identified, these active compounds could be chemically synthesized or modified to improve potency, pharmacokinetics, and brain penetration, thereby accelerating the development of more tractable drug candidates. Second, although the *C. elegans* data support the idea that PCE can operate in vivo, definitive conclusions about its therapeutic value for human AD will require validation in mammalian models. Rodent AD models, which more closely mimic the human neuropathological and cognitive decline associated with the disease, represent the next critical step. Testing PCE in these models would allow us to evaluate its effects on synaptic plasticity, learning and memory, disease progression, and other clinically relevant endpoints. Such studies should also assess PCE’s long-term safety profile, optimal dosing, and pharmacodynamics. It is also essential to determine whether PCE can cross the blood–brain barrier in sufficient quantities to exert direct central nervous system effects, a key consideration for any candidate AD therapeutic. Third, while our findings highlight the importance of mitophagy, it remains unclear how PCE interacts with other cellular networks involved in proteostasis and neuroprotection. The interplay between the ubiquitin–proteasome system, lysosomal function, chaperone-mediated autophagy, and mitochondrial biogenesis pathways could influence the extent and durability of PCE’s protective effects. In-depth mechanistic studies, employing transcriptomics, proteomics, and metabolomics, could reveal how PCE reprograms cellular homeostasis at multiple levels. Such data would not only illuminate the PCE’s comprehensive mechanism of action but might also identify additional targets or pathways that can be synergistically modulated.

At present, although some compounds have been shown to reduce Tau burden or enhance mitophagy, few address these two pathology drivers concurrently and as robustly as PCE appears to do. Furthermore, the discovery-based framework, screening natural medicine extracts for both autophagy induction and Tau cytotoxicity reduction, could serve as a template for future drug discovery efforts. Integrating this approach with high-throughput screening platforms, automated imaging systems, and machine learning algorithms could rapidly identify other natural products or synthetic analogs that share or exceed PCE’s protective potential. Another consideration is whether PCE could complement existing or emerging AD therapies. For example, could PCE enhance the efficacy of anti-amyloid agents or synergize with interventions targeting neuroinflammation or tau kinases? Given its dual actions on Tau phosphorylation/aggregation and mitochondrial quality control, PCE may offer a unique combinatorial advantage in multimodal AD therapy.

While this study points toward a novel therapeutic avenue, it is also crucial to acknowledge that developing a successful disease-modifying therapy for AD is inherently challenging. Many strategies that show promise in preclinical models fail in clinical trials due to issues of complexity, heterogeneity of patient populations, and lack of translational fidelity between models and human pathology. The complexity inherent in PCE’s composition and the need for further fractionation and testing may prolong the path to clinical application. Nonetheless, our findings establish a strong foundation from which to pursue these goals. Finally, the positive outcomes of this study support the broader notion that harnessing natural products and traditional medicines can reveal unique therapeutic strategies for complex diseases like AD. *Pulsatilla chinensis*, historically used in TCM to address conditions associated with inflammation and infection, now appears to hold promise in a neurodegenerative context. This highlights the value of reconsidering traditional medicinal plants as a reservoir of untapped pharmacological potential. By systematically integrating ethnopharmacological knowledge, modern biochemical techniques, and rigorous disease modeling, we can uncover previously unrecognized activities and bring them closer to the clinic.

## Conclusion

In conclusion, this study demonstrates that PCE exerts multifaceted neuroprotective effects by reducing Tau phosphorylation and aggregation, enhancing mitophagy, and preserving mitochondrial function. Through simultaneous regulation of Tau proteostasis and mitochondrial quality control, PCE alleviates oxidative stress and restores cellular homeostasis. These findings highlight PCE as a promising natural therapeutic candidate for AD and provide a foundation for future work to identify its active components and validate efficacy in advanced models.

## Supplementary Information

Supplementary file 1.

## Data Availability

No datasets were generated or analysed during the current study.

## Abbreviations

3-MA: 3-Methyladenine
AD: Alzheimer's disease
ANOVA: A one-way analysis of variance
ATCC: American type culture collection
Baf: Bafilomycin A1
*C. elegans*: *Caenorhabditis elegans*
CGC: *C. elegans* Genetics Center
CID: Collision-induced dissociation
DHE: Dihydroethidium
DMEM: Dulbecco’s Modified Eagle Medium
*E. coli*: *Escherichia coli*
FBS: Fetal bovine serum
FUDR: 5-Fluoro-2′-deoxyuridine
MMP: Mitochondrial membrane potential
MTT: 3-(4,5-Dimethylthiazol-2-yl)-2,5-diphenyltetrazolium bromide
NGM: Nematode growth medium
PBS: Phosphate-buffered saline
PCE: *Pulsatilla chinensis* Extract
PI: Propidium iodide
p-Tau: Phosphorylated tau
PVDF: Polyvinylidene fluoride
Rap: Rapamycin
RNAi: RNA interference
ROS: Reactive oxygen species
SDS-PAGE: Sodium dodecyl-sulfate polyacrylamide gel electrophoresis
SEM: Standard error of the mean
TBST: Tris-buffered saline with 0.1% Tween-20
TCM: Traditional Chinese medicine
TMRM: Tetramethylrhodamine methyl ester
UHPLC: Ultra-high performance liquid chromatography
α-MEM: Alpha minimum essential medium
