# Supplementary material for: Targeted degradation of aberrant Tau for the discovery of Pulsatilla chinensis in Alzheimer’s disease
Source: Chin Med. 2026 May 20;21:137. doi: 10.1186/s13020-025-01276-x (PMC13188461; doi:10.1186/s13020-025-01276-x)
Supplement: Supplementary file 1 [file 13020_2025_1276_MOESM1_ESM.docx]

**Supplementary materials**

**Targeted degradation of aberrant Tau for the discovery of *Pulsatilla chinensis* in Alzheimer's Disease**

Lan Deng^a,†^, Can Yin^a,†^，Xiaogang Zhou^a,†^, Chi Feng^a,†^, Jianming Wu^a^, Xiaobing An^a^,Jianing Mi^c^, Lufen Huang^d^, Dalian Qin^a,^*, Lu Yu^a,^*, Ting Chen^b,^*, Anguo Wu^a,^*

*^a^Sichuan Key Medical Laboratory of New Drug Discovery and Drugability Evaluation, Department of Cardiology, the Affiliated Hospital of Southwest Medical University and Key Laboratory of Medical Electrophysiology, Southwest Medical University, School of Pharmacy, Luzhou, China, 646000. denglan910@163.com (Lan Deng); yincan200011@163.com (Can Yin); zxg@swmu.edu.cn (Xiaogang Zhou); fengafeng99@163.com (Chi Feng); jianmingwu@swmu.edu.cn (Jianming Wu); an3358825273@163.com(Xiaobing An); zxg@swmu.edu.cn (Xiaogang Zhou); dalianqin@swmu.edu.cn (Dalian Qin)；yulu863@swmu.edu.cn (Lu Yu); wuanguo@swmu.edu.cn (Anguo Wu).*

*^b^School of Pharmaceutical Sciences, China-Pakistan International Science and Technology Innovation Cooperation Base for Ethnic Medicine Development in Huna Province, Hunan University of Medicine, Huaihua, China, 418000. chenting@hnmu.edu.cn (Ting Chen).*

*^c^State Key Laboratory of Traditional Chinese Medicine Syndrome, The Second Affiliated Hospital of Guangzhou University of Chinese Medicine, Guangzhou, Guangdong, China, 510120. mjnrhw@hotmail.com (Jianing Mi).*

*^d^Department of Pharmacy, Jining Medical University, Rizhao, Shandong, China, 276500. huanglufen0029@126.com (Lufen Huang).*

*Corresponding authors: Lu Yu (E-mail: yulu863@swmu.edu.cn), Ting Chen (E-mail: chenting@hnmu.edu.cn), and Anguo Wu (Tel.: +86 17769617417; E-mail: wuanguo1114@swmu.edu.cn).

^†^ Authors contribute to equal work.


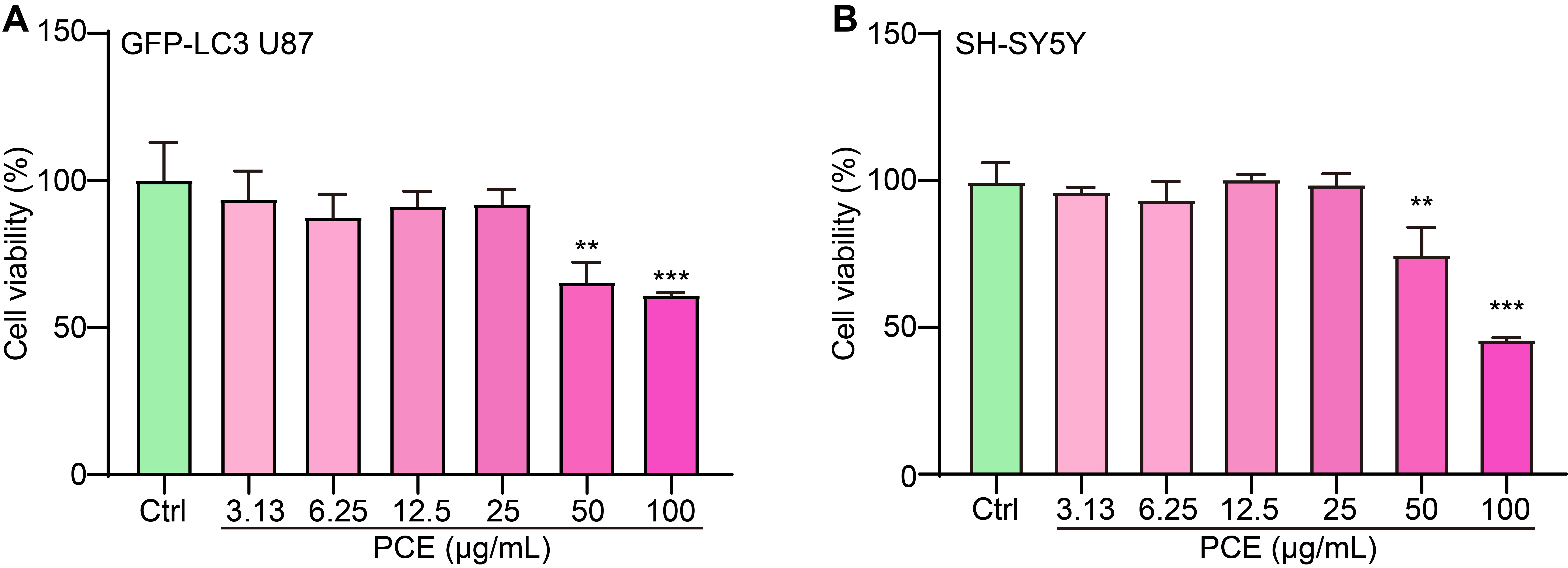


**Figure S1.** Dose-dependent effects of PCE on cell viability in SH-SY5Y and U87 cell lines. (A) Cell viability of SH-SY5Y cells treated with increasing concentrations of PCE (3.13–100 μg/mL). (B) Cell viability of GFP-LC3 U87 cells treated with increasing concentrations of PCE (3.13–100 μg/mL). Data are presented as mean ± SEM; ***p* < 0.01 and ****p* < 0.001 compared to Ctrl.


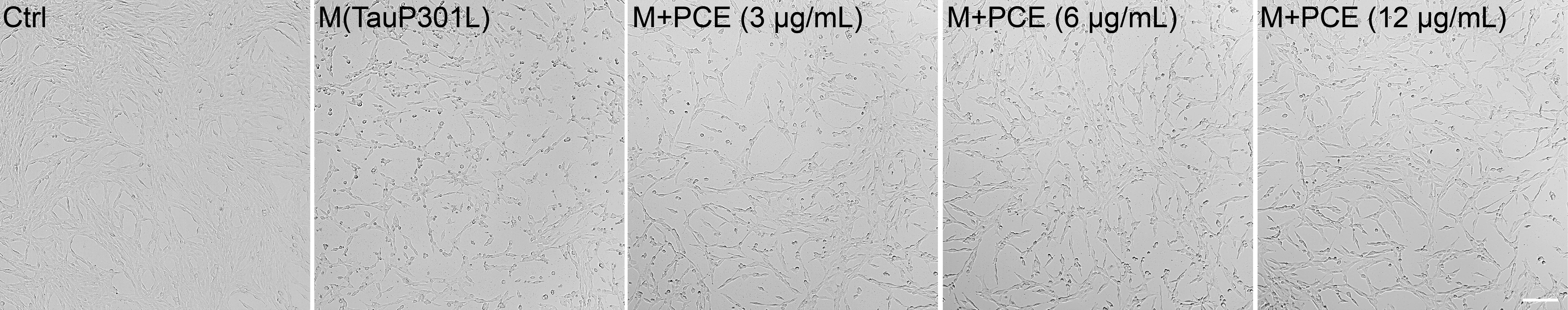


**Figure S2.** Morphological examination of SH-SY5Y cells expressing Tau P301L treated with PCE. Representative bright-field images of SH-SY5Y cells under Ctrl conditions, mock-transfected Tau P301L, and Tau P301L-expressing cells treated with increasing concentrations of PCE (3, 6, 12 µg/mL). Magnification: 10×, scale bar: 50 μm.


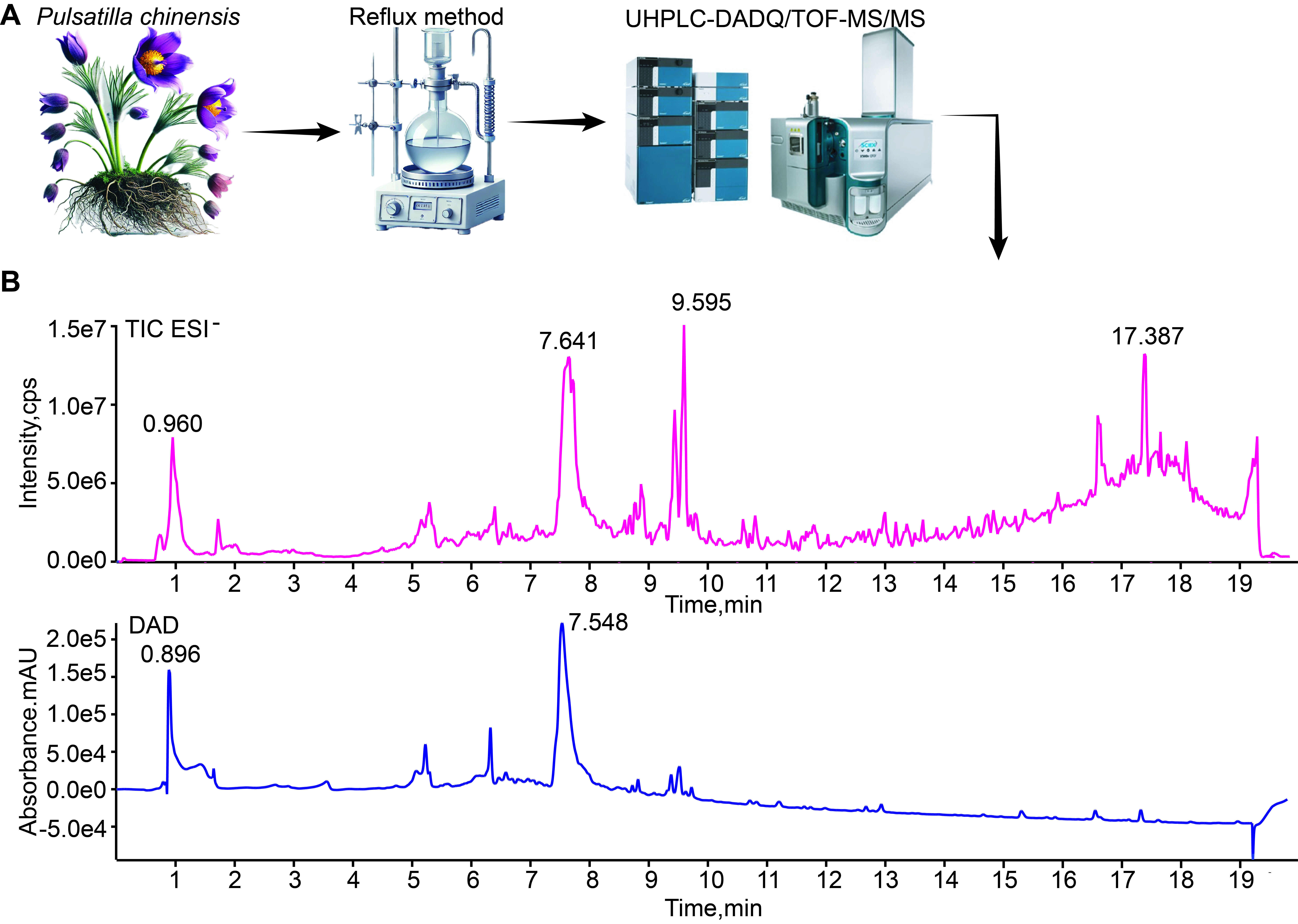


**Figure S3.** Characterization of PCE by UHPLC-DAD-Q/TOF-MS/MS. (A) Schematic representation of the extraction and analytical workflow. *Pulsatilla chinensis* plant material was processed using a reflux extraction method to obtain a crude extract. The resulting extract was subsequently analyzed by ultra-high-performance liquid chromatography coupled with diode array detection and quadrupole time-of-flight mass spectrometry/mass spectrometry (UHPLC-DAD-Q/TOF-MS/MS). (B) Representative total ion current (TIC) chromatogram recorded in negative ion mode (top) and a corresponding diode array detector (DAD) chromatogram (bottom) from the PCE.





**Figure S4.** The chemical names and molecular structures of identified compounds in PCE.


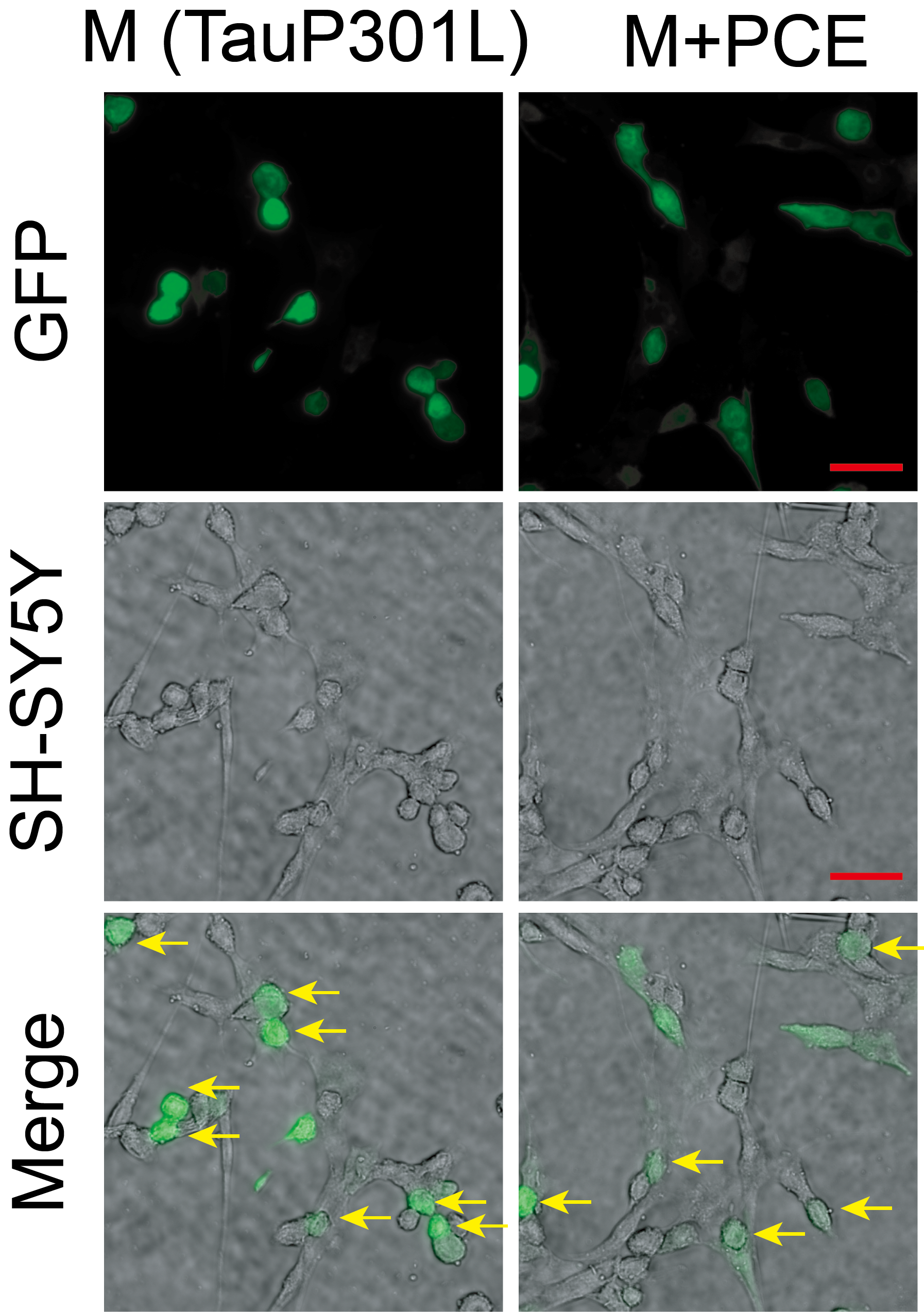


**Figure S5.** PCE inhibits Tau P301L-induced inclusion formation in SH-SY5Y cells. Representative fluorescence and bright-field merged images showing the effects of PCE on Tau inclusions in SH-SY5Y cells transfected with the Tau P301L plasmid. Yellow arrows indicate GFP-positive Tau inclusion, reflecting aberrant Tau aggregation within the cytoplasm. Magnification: 40×; scale bar = 50 μm.


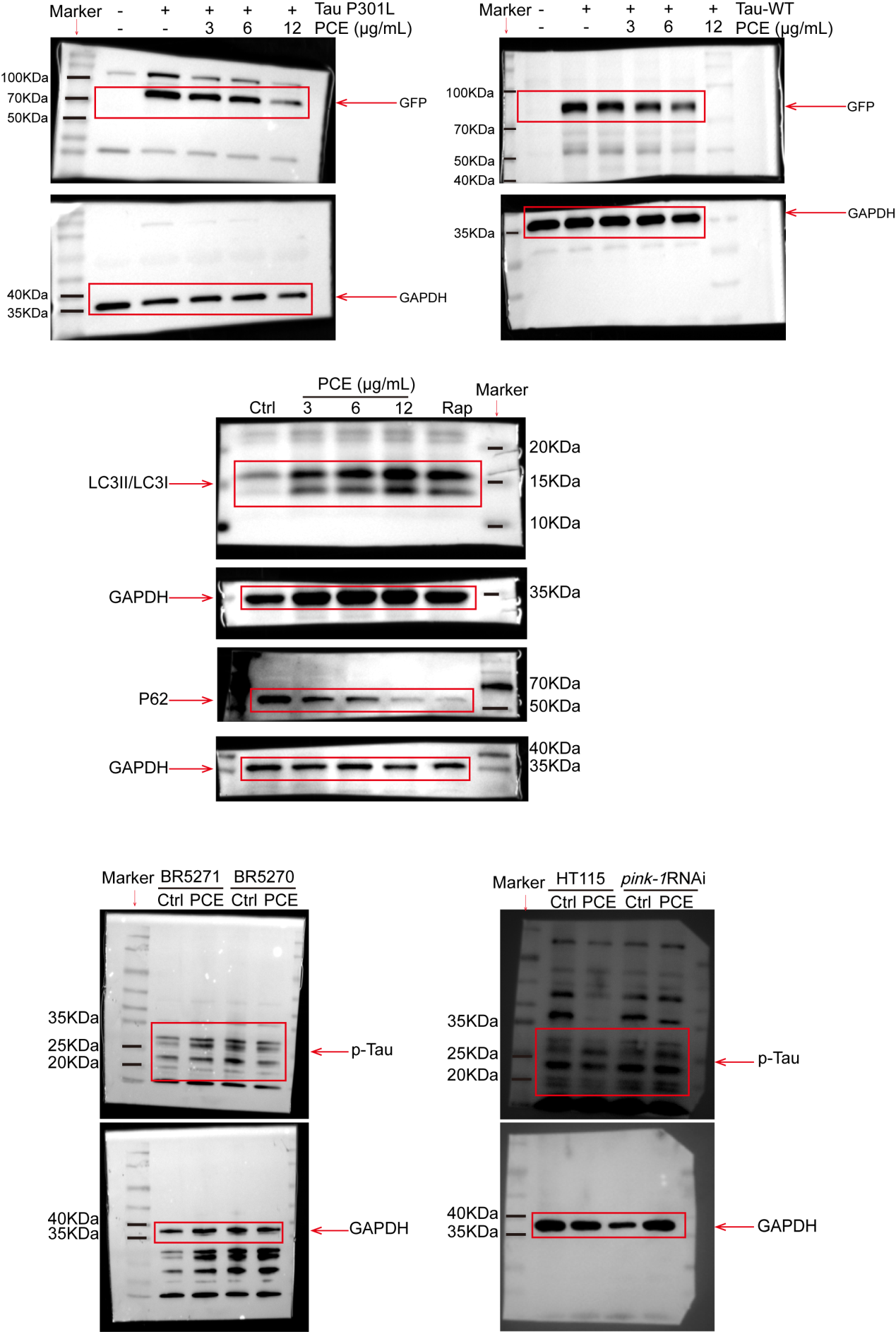


**
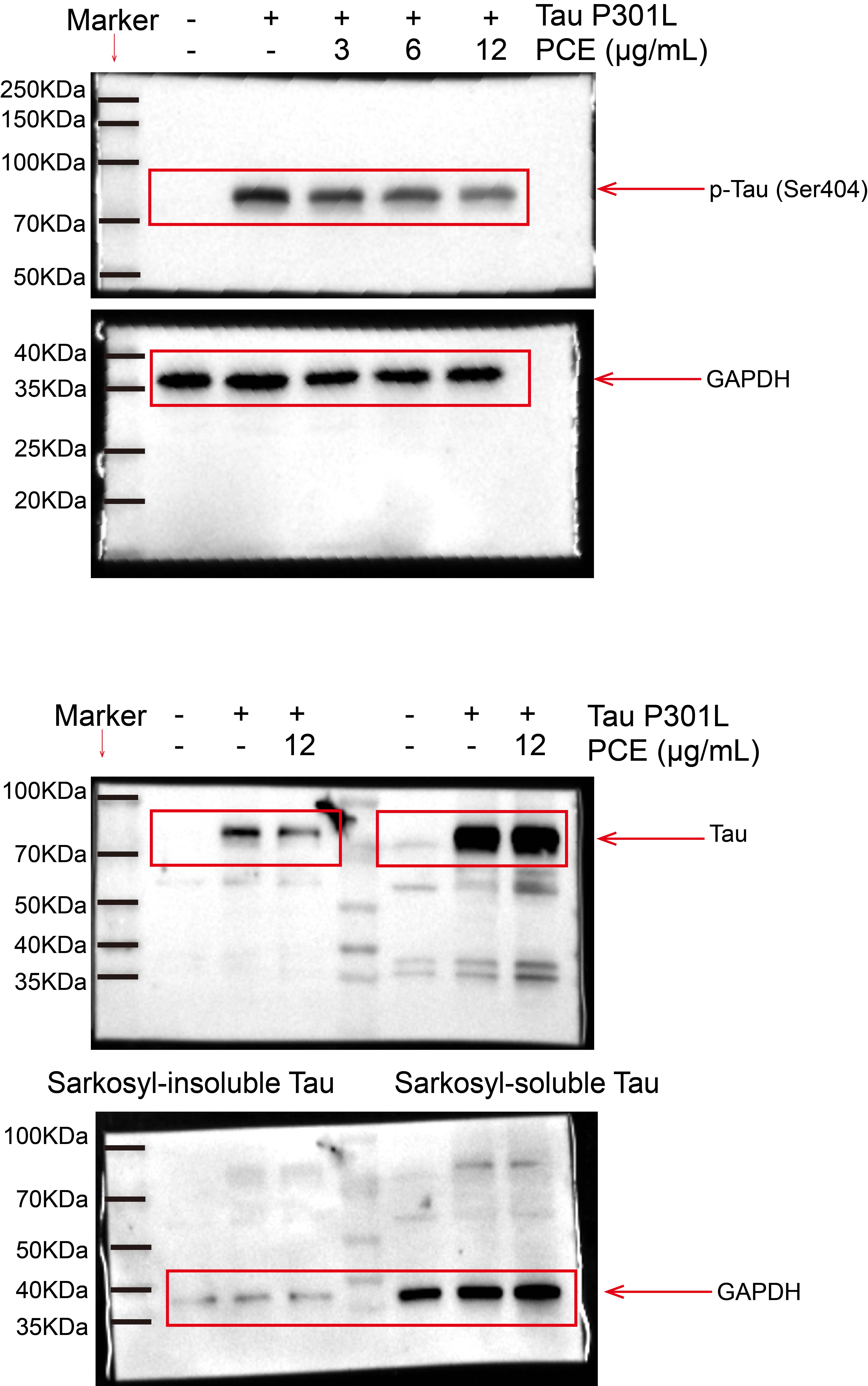
**

**Figure S6.** Full-length Western blotting images of Fig. 2A, B, I, and K


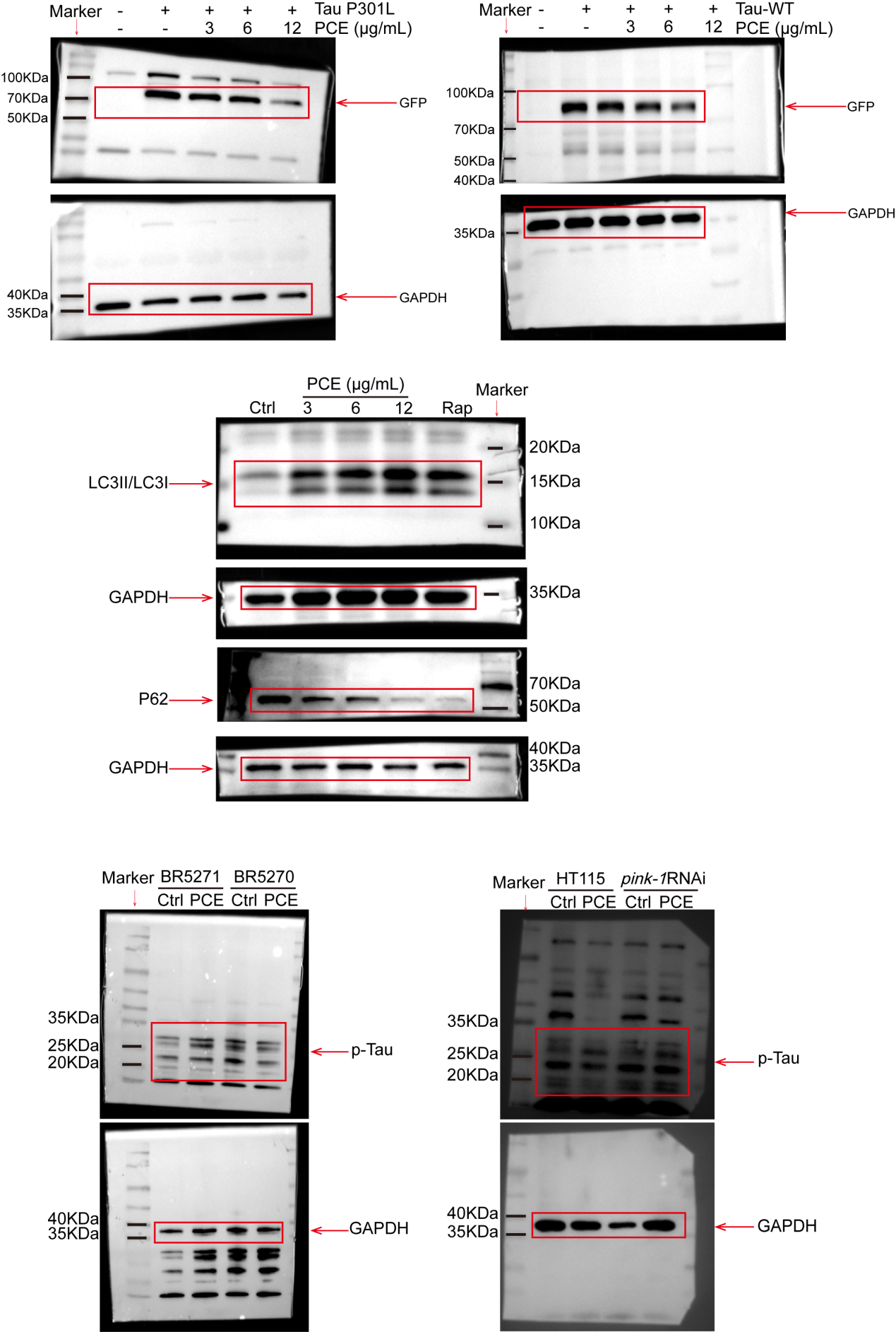


**Figure S7.** Full-length Western blotting images of Fig. 5A


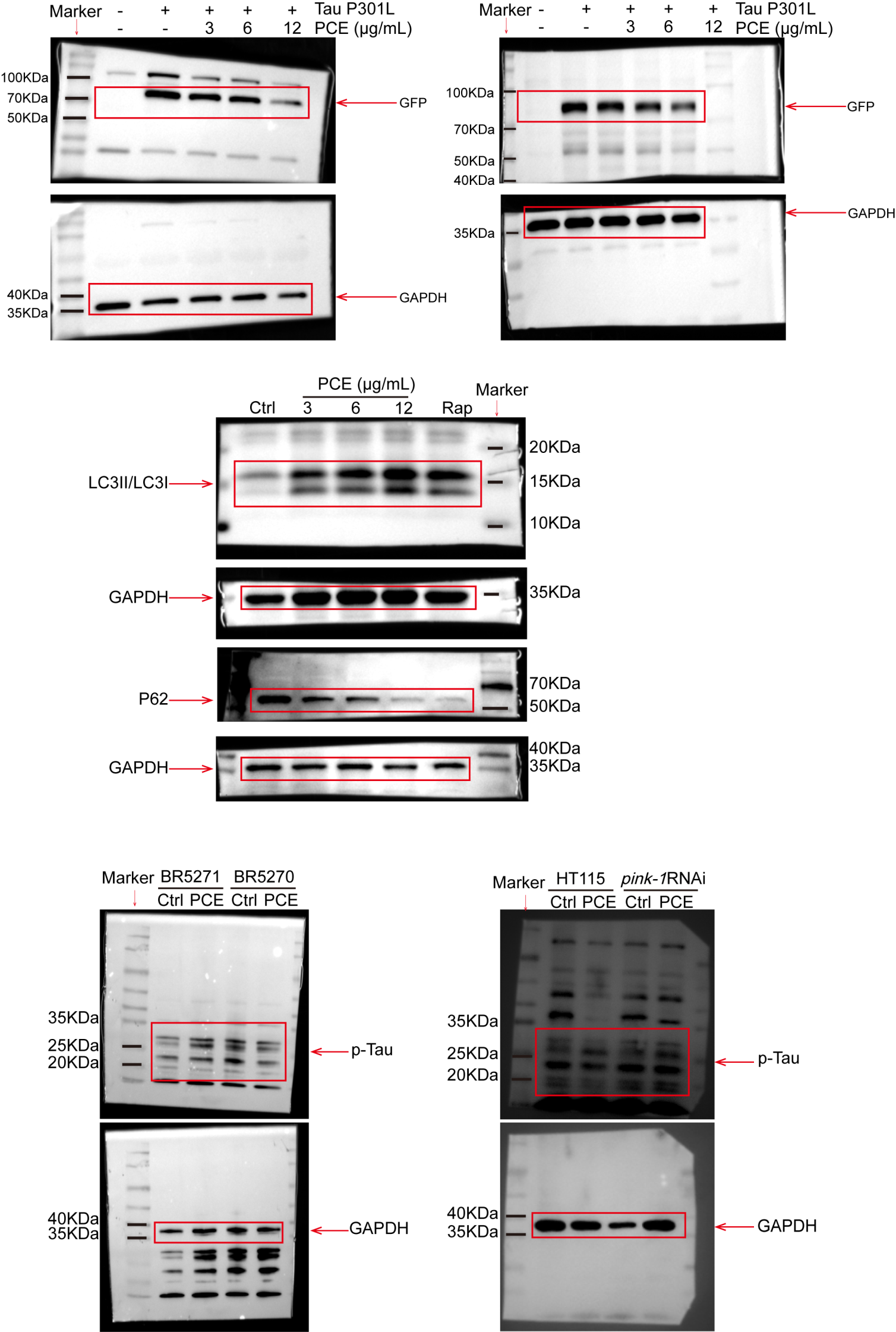


**Figure S8.** Full-length Western blotting images of Fig. 7G


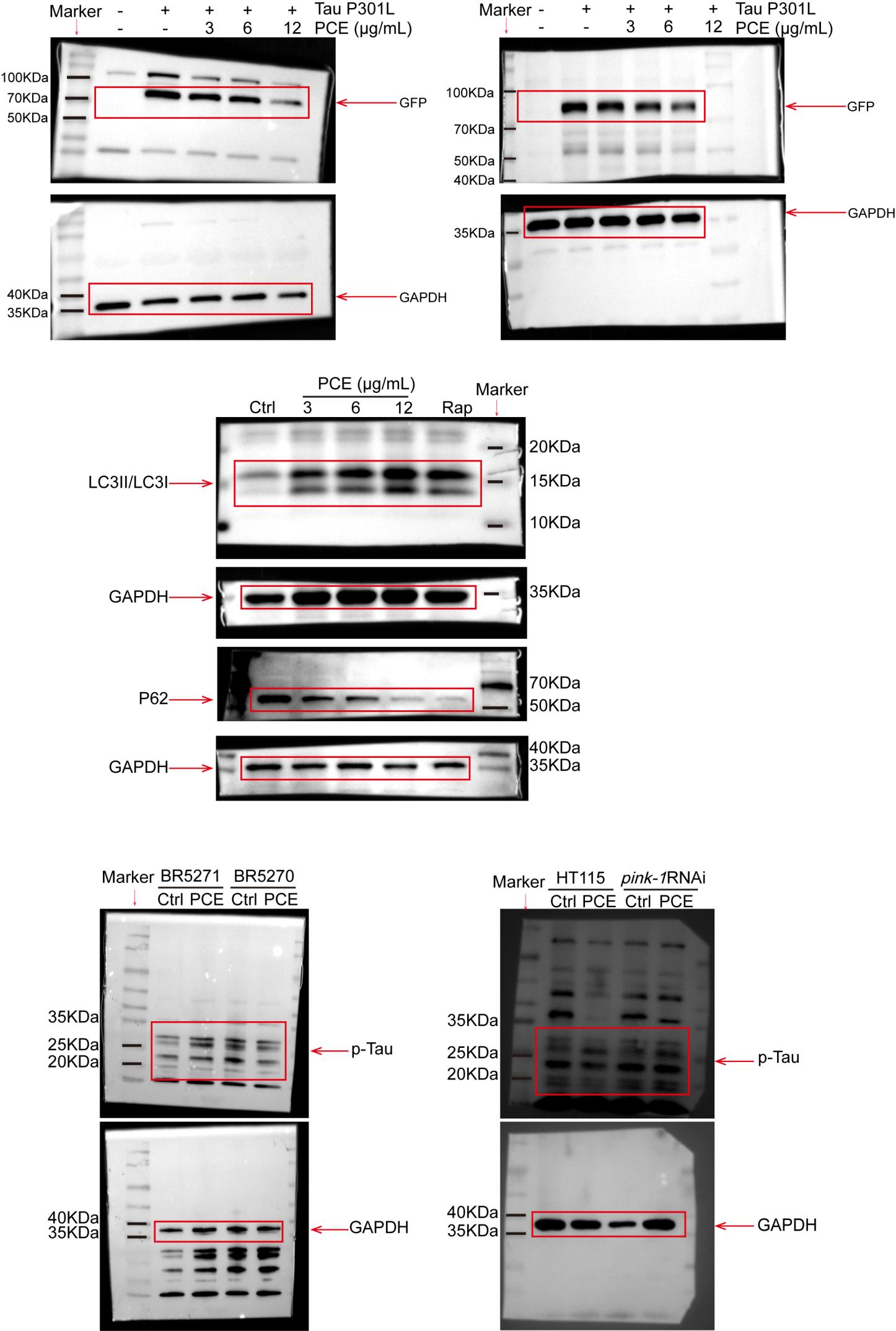


**Figure S9.** Full-length Western blotting images of **Fig. 9G**

**Table S1.** The retention time (RT), chemical name, chemical formula, molecular weight, and accurate mass of identified components in PCE.

| **No** | **RT (min)** | **Chemical name** | **Chemical formula** | **Molecular weight** | **[M-H]^-^** |
| --- | --- | --- | --- | --- | --- |
| 1 | 0.903 | Xanthotoxine | C_12_H_8_O_4_ | 216.0422 | 215.035 |
| 2 | 0.954 | Anemonin | C_10_H_8_O_4_ | 192.0422 | 191.035 |
| 3 | 5.266 | Caftaric acid | C_13_H_12_O_9_ | 312.0481 | 311.0409 |
| 4 | 7.687 | Cichoric acid | C_22_H_18_O_12_ | 474.0798 | 473.0725 |
| 5 | 8.96 | Anemoside B4 | C_59_H_96_O_26_ | 1220.6189 | 1219.6117 |
| 6 | 9.45 | Beesioside Q | C_65_H_106_O_30_ | 1366.6768 | 1365.6696 |
| 7 | 9.591 | Pulsatilloside E | C_65_H_106_O_31_ | 1382.6718 | 1381.6645 |
| 8 | 9.719 | Cussosaponin C | C_59_H_96_O_25_ | 1204.624 | 1203.6168 |
| 9 | 9.796 | Pulsatilloside D | C_59_H_96_O_27_ | 1236.6139 | 1235.6066 |
| 10 | 10.825 | Pulchinenoside B | C_53_H_86_O_22_ | 1074.541 | 1073.5538 |
| 11 | 11.028 | Pulsatilloside C | C_48_H_78_O_18_ | 942.5188 | 941.5115 |
| 12 | 11.513 | Kalopanaxsaponin H | C_47_H_76_O_17_ | 912.5082 | 911.501 |
| 13 | 11.713 | α-hederin | C_41_H_66_O_12_ | 750.4554 | 749.4439 |
| 14 | 11.736 | Pulsatilloside A | C_35_H_56_O_8_ | 603.3902 | 604.3975 |
| 15 | 12.069 | Anemoside A3 | C_41_H_66_O_12_ | 750.4554 | 749.4482 |
| 16 | 12.758 | Betulinicacid3b-O-a-L-rhamnopyranosyl-(1→2)-[b-D-glucopyranosyl (1→4)]-a-L-arabinopyranoside | C_47_H_76_O_16_ | 896.5133 | 895.5061 |
| 17 | 13.451 | Leontoside B | C_41_H_66_O_13_ | 766.4503 | 765.4431 |
| 18 | 13.534 | Tauroside C | C_41_H_66_O_11_ | 734.4605 | 733.4532 |
| 19 | 14.305 | 23-hydroxybetulinic acid | C_30_H_48_O_4_ | 472.3552 | 471.348 |
| 20 | 14.424 | Pulsatillic acid | C_30_H_46_O_4_ | 470.3396 | 469.3323 |
| 21 | 17.092 | Betulinic acid | C_30_H_48_O_3_ | 456.3603 | 455.3517 |
| 22 | 17.14 | Ursolic acid | C_30_H_48_O_3_ | 456.3603 | 455.3531 |
